# Symposia Abstracts

**DOI:** 10.1080/24740527.2017.1329303

**Published:** 2017-05-22

## Opioids for chronic pain - Finding the balance again

Roman D. Jovey*, Dawn Petit, and Lydia Hatcher

CPM Centres for Pain Management, Mississauga, ON, Canada

**CONTACT** Roman D. Jovey drjovey@bell.net


^*^Symposium Chair


© 2017 Roman D. Jovey, Dawn Petit, and Lydia Hatcher. Published with license by Taylor & Francis.

This is an Open Access article distributed under the terms of the Creative Commons Attribution License (http://creativecommons.org/licenses/by/3.0/), which permits unrestricted use, distribution, and reproduction in any medium, provided the original work is properly cited. The moral rights of the named author(s) have been asserted.

Opioids have been prescribed long-term for patients with CNCP for over 20 years. In spite of the absence of long-term RCT evidence for efficacy, patients and providers have endorsed significant long-term benefits for a subset of patients. However the ongoing publicity regarding misuse, abuse, addiction and the increasing toll of opioid-related deaths has resulted in calls for regulatory pressure to reduce total opioid prescribing for all patients.

This symposium will update participants on the problem of opioid misuse and its impact on people with pain, discuss potential improved screening criteria to optimize future patient selection and will summarize the key recommendations from the 2017 Canadian Opioid Guideline recommendations for safe and effective opioid prescribing.

## Speaker 1 Abstract Title: *Opioids for CNCP - Challenging Assumptions, Improving Patient Selection*

**Speaker 1 Name**: Roman D. Jovey

**Speaker 1 Affiliation**: CPM Centres for Pain Management, Mississauga, ON, Canada

Over the past 2 years, Canadian clinicians have been inundated with reports in the media, from governments and from regulators regarding the “Opioid Crisis”. The most visible problem has been the rising toll of opioid related deaths. Since a number of studies have associated increasing opioid prescribing for pain with increased deaths, one of the proposed solutions has been to reduce overall opioid prescribing for pain. The premise is that a high percentage of prescribed opioids are being misused, resulting in increasing rates of addiction and contributing to a high risk of death from opioid overdose. This presentation will use existing published data to challenge some of these beliefs and propose alternatives.

One of these alternatives could be to improve patient selection in order to maintain the availability of opioids for those who benefit while reducing the risks for others. The speaker will propose screening tools that go beyond simple screening of misuse risk.

## Speaker 2 Abstract Title: *The Impact of the Opioid Crisis on People With Pain*

**Speaker 2 Name**: Dawn Petit

**Speaker 2 Affiliation**: President, Pain Society of Alberta, West Grove Primary Care Network, Spruce Grove, Alberta, Canada

From her perspectives as a health professional, a person with pain, and the president of a provincial pain society, the speaker will share her observations on the impact of the societal opioid crisis on people with pain.

## Speaker 3 Abstract Title: *The Canadian Opioid Guidelines 2017 - Something Old, Something New*

**Speaker 3**: Lydia Hatcher

**Speaker 3 Affiliation**: Chief of Family Medicine, St. Joseph’s Healthcare Hamilton, Associate Clinical Professor McMaster University, Hamilton, Ontario, Canada

The first version of the Canadian Guideline for Safe and Effective Use of Opioids for the Treatment of Chronic Non-Cancer Pain was published in 2010. At the time it was recognized as one of the most rigorously developed opioid guidelines in the world.

Now, seven years later, evolving evidence and clinical experience and the increasing concern regarding the potential societal harms of prescribed opioids have made an update to the guidelines essential.

This presentation will review the process and some of the key recommendations from the 2017 update and focus on the practical implications of any changes.

## Learning Objectives

1. Develop a practical strategy to reduce the risks of opioids by optimizing the selection of patients with chronic pain who might benefit from opioids

2. Discuss the potential impact on people with pain of recent government and regulatory strategies to reduce opioid prescribing

3. Describe the impact of some of the significant changes in the 2017 Canadian Opioid Guidelines

## Calcium regulation and targets for pain and analgesia

Bradley Kerr^a*^, Michael Gold^b^, and Gerald Zamponi^c^

^a^Department of Pharmacology, University of Alberta, Edmonton, Alberta, Canada; ^b^University of Pittsburgh, Department of Neurobiology, Pittsburgh, PA, USA; ^c^Department of Physiology and Pharmacology, Hotchkiss Brain Institute and Alberta Children’s Hospital Research Institute, Cumming School of Medicine, University of Calgary, Calgary, Alberta, Canada

**CONTACT** Bradley Kerr bradley.kerr@ualberta.ca


^*^Symposium Chair


© 2017 Bradley Kerr, Michael Gold, and Gerald Zamponi. Published with license by Taylor & Francis.

This is an Open Access article distributed under the terms of the Creative Commons Attribution License (http://creativecommons.org/licenses/by/3.0/), which permits unrestricted use, distribution, and reproduction in any medium, provided the original work is properly cited. The moral rights of the named author(s) have been asserted.

Intracellular Ca2+ is essential for a variety of cellular processes, and as a result, intracellular Ca2+ levels are tightly regulated. This symposium will focus on distinct components of Ca2+ signaling and regulation in the presence of absence of tissue injury and the evidence in support of these components as mechanisms of pain associated with tissue injury as well as targets for novel therapeutic interventions. Michael Gold will focus on the role of different Ca2+ regulatory mechanisms in the response to inflammation and nerve injury. Bradley Kerr will present data on mitochondrial dysfunction and oxidative stress in the MS model that is likely mediated through dysfunction of the Ca2+ handling. Finally, Gerald Zamponi will discuss recent data on the role of T-type voltage-gated Ca2+ channels in both nociceptive processing and as potential targets for novel therapeutic interventions

## Speaker 1 Abstract Title: *Impact of the type of injury on Ca2+ regulation on subpopulations of nociceptive afferents*

**Speaker 1 Name**: Michael Gold

**Speaker 1 Affiliation**: University of Pittsburgh, Department of Neurobiology, Pittsburgh, PA, USA

**Speaker 1 Abstract**” It has long been appreciated that voltage-gated Ca2+ channels in sensory neurons are viable therapeutic targets for the treatment of pain. These channels are critical for transmitter release, and therefore enable the dynamic regulation of afferent input to the central nervous system. However, their therapeutic utility is complicated by observations suggesting that dysregulation of these channels also contributes to injury-induced pain and hypersensitivity. Interestingly, while these channels play an essential role in the initial influx of Ca2+ associated with afferent activity, a number of other Ca2+ regulatory proteins play a dominant role in the control of both the magnitude and duration of the evoked intracellular Ca2+ transient. Recent evidence indicates that several of these Ca2+ regulatory proteins are altered in response to tissue injury resulting in significant changes in the dynamics of activity evoked Ca2+ transient. Because these changes may also contribute to the pain and hypersensitivity associated with tissue injury via direct influences on ion channel activity and transmitter release as well as indirectly, via changes in gene expression, these Ca2+ regulatory mechanisms may also have therapeutic utility. Michael Gold will discuss recent data from models of inflammation and nerve injury highlighting the distinct pattern of changes in Ca2+ regulation observed in these injury models, the different subpopulations of afferents impacted, and the underlying mechanisms that are not only specific to the type of injury by the afferent subpopulation. He will discuss these data in the context of potential therapeutic targets.

## Speaker 2 Abstract Title: *Transient inflammation in the PNS leads to oxidative stress and calcium dysregulation in MS associated pain states*

**Speaker 2 Name**: Bradley Kerr

**Speaker 2 Affiliation**: Department of Pharmacology, University of Alberta, Edmonton, Alberta, Canada

**Speaker 2 Abstract**: The neuroinflammatory disease Multiple Sclerosis (MS) is most often associated with impairments to motor function due to the demyelination of axon tracts in the CNS. Over the past decade however, there has been a greater awareness around the fact that MS is also associated with significant changes in sensory function including a high incidence of neuropathic pain in patients with the disease. While it has been assumed that pain in MS originates in the CNS, changes in response to inflammation at peripheral sites such as the dorsal root ganglion (DRG) may also contribute to these abnormal pain states. We have been conducting studies using a mouse model of MS, experimental autoimmune encephalomyelitis (EAE), and have observed a significant, although transient, inflammatory response in the DRG of mice with EAE. Our data suggests that while the inflammatory response in the DRG is short lived, long term changes in the cell bodies emerge in response to this inflammation including altered expression of key regulators of intra cellular calcium. These changes can have consequences for the structural integrity of the dorsal roots projecting into the spinal cord and result in an increase in the excitability and output of the cells in response to stimulation. Altered neuronal excitability was most prominent in large diameter, presumptive low threshold, Aβ cells and this likely underlies the emergence of cold and tactile allodynia-like behaviours that are observed in mice with EAE. Thus, our data reveals that neuropathic pain in MS may not be of an exclusive CNS origin.

## Speaker 3 Abstract Title: *Targeting ubiquitination of calcium channels – a novel therapeutic strategy for pain*

**Speaker 3 Name**: Gerald Zamponi

**Speaker 3 Affiliation**: Department of Physiology and Pharmacology, Hotchkiss Brain Institute and Alberta Children’s Hospital Research Institute, Cumming School of Medicine, University of Calgary, Calgary, Alberta, Canada

**Speaker 3 Abstract**: T-type calcium channels of the Cav3.2 subtype are expressed in afferent fibers and the spinal dorsal horn, where they contribute to the release of neurotransmitters and the regulation of neuronal firing properties. In a number of chronic pain conditions, these channels are dysregulated such that their whole cell current densities are enhanced. Conversely, blocking Cav3.2 channels or knocking down their expression results in analgesia. We have shown that this aberrant upregulation is due to an association of the deubiquitinase USP5 with the Cav3.2 channel domain III-IV linker. Under chronic pain conditions, USP5 expression is enhanced; leading to removal of ubiquitin groups from the channel and thus increased Cav3.2 levels in the plasma membrane. Transcutaneous optogenetic activation of TRPV1 expressing C fibers, but not Thy-1 expressing cells is sufficient to cause an activity-dependent enhancement of USP5 expression, indicating that this pathway is activated in specific subtypes of afferent fibers. Knockdown of USP5, or interfering with its association with the channel via cell permeant disruptor peptides protects from inflammatory, neuropathic, visceral and diabetic pain hypersensitivity in mice. Along these lines, small organic molecules that target this association are similarly effective, thus potentially providing a novel therapeutic approach for treating a wide range of painful conditions.

## Learning Objectives

1. Review the diversity of Ca2+ regulatory mechanisms

2. Illustrate the differential impact of the type of injury on the dysregulation of intracellular Ca2+ among subpopulations of neurons

3. Highlight evidence in support novel therapeutic targets for the treatment of pain

## Spinal Cord Stimulation for the treatment of neuropathic pain

Lutz Weise^a,*^, Cecile C. de Vos^b^, and Jeffrey Kramer^c^

^a^Division of Neurosurgery, Dalhousie University, Halifax, Nova Scotia, Canada; ^b^Medisch Spectrum Twente hospital (NL), Enschede, Nederland and McGill University (CA), Montreal, Quebec, Canada; ^c^St. Jude Medical, Saint Paul, Minnesota, USA

**CONTACT** Lutz Weise Lutz.Weise@nshealth.ca


^*^Symposium Chair


© 2017 Lutz Weise, Cecile C. de Vos, and Jeffrey Kramer. Published with license by Taylor & Francis.

This is an Open Access article distributed under the terms of the Creative Commons Attribution License (http://creativecommons.org/licenses/by/3.0/), which permits unrestricted use, distribution, and reproduction in any medium, provided the original work is properly cited. The moral rights of the named author(s) have been asserted.

Spinal cord stimulation (SCS) for the treatment of neuropathic pain has been available for over forty years and its efficacy has been demonstrated for chronic intractable pain of various aetiologies.

However, technological development of SCS therapy has been relatively slow and only recently several new stimulation paradigms and targets have become available, that might allow for broader application of the technology.

In this symposium we will give an overview of the current evidence for SCS therapy, the first results of several new forms of SCS and how these new stimulation approaches can be used to target specific patient populations and further personalise pain treatment.

## Speaker 1 Abstract Title: *Evidence for the analgesic effects of Spinal Cord Stimulation*

**Speaker 1 Name**: Lutz Weise

**Speaker 1 Affiliation**: Division of Neurosurgery, Dalhousie University, Halifax, Nova Scotia, Canada

**Speaker 1 Abstract**: For the past four decades electrical stimulation of the dorsal spinal cord has demonstrated to be a safe and effective therapeutic tool for relieving several chronic neuropathic pain conditions, which are generally difficult to treat with medication. Randomized controlled trials have been conducted in patients with failed back surgery syndrome, complex regional pain syndrome and diabetic neuropathy.

Spinal cord stimulation (SCS) is commonly administered via a stimulation electrode positioned over the dorsal column and by using an implantable pulse generator to deliver electrical pulses with adjustable stimulation frequency, pulse width and pulse amplitude to aim for optimal pain relief. This mode of SCS is generally accompanied by paraesthesia in the area covered by the stimulation.

## Speaker 2 Abstract Title: *What are the benefits and draw backs of new spinal cord stimulation settings?*

**Speaker 2 Name**: Cecile C. de Vos

**Speaker 2 Affiliation**: Medisch Spectrum Twente hospital (NL), Enschede, Nederland and McGill University (CA), Montreal, Quebec, Canada

**Speaker 2 Abstract**: Background: For a very long time, the paraesthesia that accompanies spinal cord stimulation (SCS) has been considered mandatory to obtain pain relief. Recently several new paraesthesia-free stimulation paradigms have been developed.

New stimulation paradigms: Conventional SCS provides continuous stimulation with frequencies of typically between 40 Hz and 100 Hz. High Frequency stimulation provides electrical pulses with a frequency of 10 kHz. Burst stimulation administers 40 times per second a burst consisting of five pulses with a frequency of 500 Hz. Both these new stimulation paradigms have been shown to suppress neuropathic pain at least as well as conventional stimulation. Burst stimulation is approved by Health Canada since June 2015, High Frequency stimulation is not yet available in Canada.

Potential benefits: Paraesthesia no longer limits the application of SCS. So different and larger body areas can be stimulated and higher stimulation settings can be used, resulting in a broader use of SCS and better and more consistent pain relief.

Potential draw backs: All these new stimulation paradigms deliver substantially more energy to the spinal cord without the patient sensing the stimulation. Patients might get over stimulated and experience side effects. In addition, we do not yet know the long term consequences of this increased energy application to the spinal cord.

## Speaker 3 Abstract Title: *What are the benefits and draw backs of new neurostimulation targets?*

**Speaker 3 Name**: Jeffrey Kramer

**Speaker 3 Affiliation**: St. Jude Medical, Saint Paul, Minnesota, USA

**Speaker 3 Abstract**: Background: The dorsal root ganglion (DRG) is located in every vertebra in the spine and comprises nerves involved in sending pain messages to the brain. Neurostimulation of the DRG is a recent development in the field of neuromodulation. This targeted neurostimulation sends mild electrical pulses to the DRG which may result in mild paraesthesia and significant reductions in pain.

Current stage of development: A neurostimulator developed to target the DRG received CE Mark in November 2011 and FDA approval in February 2016. DRG stimulation is now used in several European countries and in the US, but not yet in Canada.

Potential benefits: Twelve-month data of the ACCURATE study indicated superiority of DRG over conventional SCS across all primary endpoint analyses. The location of the DRG electrode lead prevents movement of the lead which might result in more consistent pain relief and less variability in sensation of the paraesthesia.

Potential draw backs: The novelty of the technique may initially lead to a higher overall incidence of adverse events when compared with SCS. The lack of experience with stimulation of the DRG may lead to loss of stimulation, overstimulation or difficulties in the management of complications resulting from hardware malfunction e.g. there have been reports of lead breakage during attempted lead removal.

## Learning Objectives

1. Know what types of pain can be treated effectively with spinal cord stimulation.

2. Gain an understanding of current evidence in the field of spinal cord stimulation

3. Understand the possibilities and limitations of new technological developments in neuromodulation.

## The Link between Stress and Pain Across the Lifespan: Evidence from Children, Adults, and Rodents

Melanie Noel^a,*^, Joel Katz^b^, and Loren Martin^c^

^a^Department of Psychology, University of Calgary and Alberta Children’s Hospital Research Institute, Calgary, Alberta, Canada; ^b^Department of Psychology, York University, Toronto, Ontario, Canada; ^c^Department of Psychology, University of Toronto, Toronto, Ontario, Canada

**CONTACT** Melanie Noelmelanie.noel@ucalgary.ca


^*^Symposium Chair


© 2017 Melanie Noel, Joel Katz, and Loren Martin. Published with license by Taylor & Francis.

This is an Open Access article distributed under the terms of the Creative Commons Attribution License (http://creativecommons.org/licenses/by/3.0/), which permits unrestricted use, distribution, and reproduction in any medium, provided the original work is properly cited. The moral rights of the named author(s) have been asserted.

By its very definition, pain is an emotionally distressing experience. Stress and pain systems are intrinsically tied. As an evolutionarily salient alarm signal, pain activates a stress response, which propels action. On the other hand, stress has been shown to confer risk for the development and maintenance of pain problems. The stress-pain relationship should be understood from a developmental perspective. There is compelling evidence to suggest that pain experienced early in life reprograms the HPA axis and leads to altered stress responses later in life. Moreover, early life stress has been linked to chronic pain conditions in adulthood. Among adults, those individuals with a heightened propensity to become traumatized by pain are more likely to subsequently develop chronic pain. Theorists have argued that the co-occurrence of traumatic stress and pain is mutually maintained by underlying cognitive, behavioral and neurobiological mechanisms. Moreover, heightened levels of subjective stress have been shown to impede response to pain management interventions. Better understanding of the connection between stress and pain could lead to improved, tailored treatments and address the growing epidemic of chronic pain in childhood and adulthood.

This workshop will present the current state-of-the-science on the relationship between stress and pain across childhood, adolescence, and adulthood. It will consist of a panel of clinical and basic scientists examining this topic using a variety of innovative methodologies and approaches, populations (acute and chronic pain and transition in between [post-surgical pain]), and perspectives.

## Speaker 1 Abstract Title: *The Co-occurrence of Post-Traumatic Stress and Pain in Youth with Chronic Pain*

**Speaker 1 Name**: Melanie Noel

**Speaker 1 Affiliation**: Department of Psychology, University of Calgary and Alberta Children’s Hospital Research Institute, Calgary, Alberta, Canada

**Speaker 1 Abstract**: Chronic pain in childhood and adolescence is highly prevalent, impairing, and poses a large economic burden on society that exceeds the costs of asthma and obesity. In a large epidemiological study, we showed for the first time that a variety of chronic pain conditions in adolescence are linked to higher rates of post-traumatic stress disorder (PTSD) in adolescence and adulthood (Noel et al., 2016a, PAIN). In a controlled cohort study, we revealed that youth with chronic pain and their parents reported significantly higher rates of clinically elevated PTSD symptoms as compared to pain free peers and their parents (32% vs. 8%; 20% vs. 1%, respectively). Moreover, among youth with chronic pain, higher PTSD symptoms were linked to worse pain complaints and quality of life (Noel et al., 2016b, PAIN). Using new novel data from cohorts of youth with pediatric chronic pain from the US and Canada, Dr. Noel will describe the prevalence rates of PTSD symptoms in various pediatric chronic pain populations as well as the nature of traumatic events experienced by youth and their parents. She will present data on modifiable mechanisms (e.g., hypervigilance, anxiety sensitivity, depression) underlying the stress-pain relationship in these cohorts of youth. Finally, the negative impact of PTSD on functional outcomes from diagnosis to several months later in youth seen in a tertiary-level pediatric chronic pain program will be presented. Implications for treatment tailoring and integration of other treatment modalities (trauma-focused CBT) into existing chronic pain protocols for youth will be discussed.

## Speaker 2 Abstract Title: *Sensitivity to Pain Traumatization: A Novel Construct Linking Traumatic Stress Reactions and Chronic Pain to Pain-related Disability*

**Speaker 2 Name**: Joel Katz

**Speaker 2 Affiliation**: Canada Research Chair in Health Psychology; Professor of Psychology, York University, Toronto, Ontario, Canada

**Speaker 2 Abstract**: Dr. Katz will introduce the various theories that have been proposed to explain the co-occurrence of anxiety disorders such as PTSD and chronic pain. He will illustrate one of the links between traumatic stress symptoms and pain in patients undergoing major thoracic surgery using prospective data that shows as the length of time from surgery increases, symptoms of emotional numbing become increasingly tied to pain-related disability whereas the relationship between pain intensity and pain disability becomes increasingly de-coupled. He will then provide evidence for a construct termed “sensitivity to pain traumatization” (SPT) that describes the propensity to develop anxiety-related somatic, cognitive, emotional, and behavioral responses to pain that resemble features of a traumatic stress reaction. Data from patients undergoing major surgery show that preoperative SPT scores, but not posttraumatic stress symptom scores, are significantly higher in patients with persistent pain versus those without persistent pain both before, and one-year after, surgery. These findings argue for SPT as a distinct construct separate from post-traumatic stress disorder. Finally, he will describe the development and validation of a 12-item Sensitivity to Pain Traumatization Scale.

## Speaker 3 Abstract Title: *The Modulation of Pain by Environmental Stress*

**Speaker 3 Name**: Loren Martin

**Speaker 3 Affiliation**: Canada Research Chair in Translational Pain Research; Assistant Professor, Department of Psychology, University of Toronto, Toronto, Ontario, Canada

**Speaker 3 Abstract**: The modulation of pain by stress and emotion is now widely recognized as important for adaptation. Stress, depending on its nature, duration, and intensity can exert profoundly complex changes on pain states typified by either a reduction (analgesia) or exacerbation (hyperalgesia). In humans, psychological factors such as stress, anxiety, and expectation play an important role in shaping pain perception, and in the clinic, when pain is anticipated, patients often report heightened pain sensations. Thus, behaviours associated with pain may not be intrinsic to the stimulus of pain, but may be a response to external stressors and environmental reinforcers. This talk will focus on the use of novel animal models for the study of environmentally-induced hyperalgesia and recent efforts in our lab to translate these findings to people. In particular we find that mice and people become sensitized to their environment when they have had an aversive pain experience within that environment. This sensitization persists for at least 24 hours and is only present in males of both species. In mice we can abolish environmental-specific hyperalgesia by castrating male mice or by blocking the hypothalamic-pituitary-adrenal axis (HPA) axis suggesting that testosterone and stress are necessary for this phenomenon. These models provide a new means for studying the relationship between stress and pain by examining the influence of environmental stressors.

## Learning Objectives

1. To examine the prevalence, short- and long-term functional impact, and mechanisms underlying the PTSD and pain relationship in children and adolescents with chronic pain.

2. To understand the relationships between traumatic stress reactions and chronic pain in general and as they relate to chronic postsurgical pain.

3. To highlight the importance of environmental stressors on modulating pain responses using translational approaches.

## Beyond TENS Machines and Heating Pads: Development and application of advanced technologies in pain prevention, assessment and management

Thomas Hadjistavropoulos^a,*^, Catherine Mercier^b^, and Alex Mihailidis^c^

^a^Department of Psychology and Centre on Aging and Health, University of Regina, Regina, Saskatchewan, Canada; ^b^Centre interdisciplinaire de recherche en réadaptation et en intégration sociale (CIRRIS), Université Laval, Quebec, Canada; ^c^AGE-WELL NCE Inc, Toronto Rehab Institute-UHN /University of Toronto, Toronto, Ontario, Canada

**CONTACT** Thomas Hadjistavropoulos hadjistt@uregina.ca


^*^Symposium Chair


© 2017 Thomas Hadjistavropoulos, Catherine Mercier, and Alex Mihailidis. Published with license by Taylor & Francis.

This is an Open Access article distributed under the terms of the Creative Commons Attribution License (http://creativecommons.org/licenses/by/3.0/), which permits unrestricted use, distribution, and reproduction in any medium, provided the original work is properly cited. The moral rights of the named author(s) have been asserted.

The goal of this symposium is to familiarize participants with development, research and applications related to cutting edge technologies for pain prevention, assessment and management. These technologies include: a) applications of computer vision in pain assessment; b) virtual reality in the assessment and treatment of patients with conditions such as non-specific low back pain and complex regional pain syndrome; c) virtual reality in the treatment of conditions such as phantom limb pain; and d) automated and artificial intelligence systems in the prevention of painful injury.

## Speaker 1 Abstract Title: *Using virtual reality to provide altered feedback about movements: new avenues for assessment and treatment in chronic pain populations*

**Speaker 1 Name:** Catherine Mercier

**Speaker 1 Affiliation:** Centre interdisciplinaire de recherche en réadaptation et en intégration sociale (CIRRIS), Université Laval, Quebec, Canada

**Speaker 1 Abstract:** The idea of using virtual reality (VR) to treat chronic pain emerged from the observation made in amputees that viewing a virtual limb moving, achieved by looking at the reflection of the intact arm in a mirror box, can induce sensations of movement in the phantom limb and alleviate phantom limb pain. Mirror therapy or derived approaches are now being used in a variety of clinical populations, the commonalties being that the patient is always exposed to a ‘virtual’ representation of his moving missing/injured body part while imagining/attempting to reproduce the same movement. Compared to a mirror box, VR provides more flexibility, offering for example the possibility to replace both limbs for individuals with bilateral injury or to precisely control the movement parameters. It also allows for virtual/augmented feedback about body part movements in patients with no or very little residual control of their limbs. That is, a simple movement or physiological signal can be used to trigger/control a pre-recorded movement to create completely artificial feedback. Alternatively, in individuals able to move, VR can be used to manipulate the feedback provided to a patient about his or her own movement in a subtler manner. Examples will be presented for each of these two different types of VR applications: (1) a “cheating mirror” to assess and train patients with low back pain or complex regional syndrome; (2) self-paced virtual walking in patients with spinal cord injury and neuropathic pain.

## Speaker 2 Abstract Title: *Development of Clinically Useful, Inexpensive and Practical Technologies to Monitor Pain Behaviour in Advanced Dementia*

**Speaker 2 Name:** Thomas Hadjistavropoulos

**Speaker 2 Affiliation:** Department of Psychology and Centre on Aging and Health, University of Regina, Regina, Saskatchewan, Canada

**Speaker 2 Abstract:** Pain is undertreated among long-term care (LTC) residents with dementia. This is largely because residents with advanced dementia have severe limitations in ability to communicate the subjective state of pain. Well validated observational assessment methods to evaluate pain in these populations are available but these methods are not used on a regular basis because of limitations in resources and staffing. To address the problem of limited human resources for ongoing pain assessment and monitoring, our group (that includes computer vision experts and is supported by the Canadian Institutes of Health Research and the AGE WELL Network of Centers of Excellence) has conducted extensive work toward the development of an inexpensive, unobtrusive, automated vision system capable of monitoring pain behaviours in individuals with dementia as they go about their daily routines. The nature of this extensive development work will be outlined as well the next steps toward implementation and evaluation in front-line clinical settings.

## Speaker 3 Abstract Title: *Use of advanced technologies in the prevention of painful injuries in at-risk frail populations*

**Speaker 3 Name:** Alex Mihailidis

**Speaker 3 Affiliation:** AGE-WELL NCE Inc, Toronto Rehab Institute-UHN /University of Toronto, Toronto, Ontario, Canada

**Speaker 3 Abstract:** Injuries that result from slips and falls often have severe consequences for older adults, including hospitalization, chronic pain, and overall poor long-term outcomes. Currently, precise identification of older persons who are at an increased risk for painful injury is difficult and tends to be based on unreliable clinical measures that can only be administered by trained professionals in clinical settings. In recent years we have seen the development of new technologies that can monitor older adults in day-to-day settings (e.g. in their own homes) and characterize key parameters such as walking speed, changes in gait, and other indicators that may predict if an older adult is at risk for a fall. In addition, new technologies, that can automatically detect a fall and can call for help in order to attain the necessary assistance for the injured person as quickly as possible, have been developed. This presentation will feature new technologies developed and evaluated at the Toronto Rehab Institute-UHN. Results that have been obtained to support their efficacy will also be presented. These technologies include: 1) an automated gait analysis tool for monitoring changes in older adults’ gait in their own homes; 2) a fall detection system that uses artificial intelligence to automatically detect falls; and 3) a fall prediction system that can assess key parameters to determine if an older adult is at risk of sustaining a painful fall-related injury in the near future.

## Learning Objectives

1. To provide an overview of cutting edge advanced technologies designed to prevent pain, assess and treat pain

2. To familiarize participants with the scientific process leading to the development and evaluation of such technologies

3. To discuss use of these technologies in real life settings such as health care facilities

## The Pain Management Barrier: Let’s break on through to the other side

Ruth Dubin^a,*^, Maureen Allen^b^, and Terry Bremner^c^

^a^Queens University, Kingston, Ontario, Canada and Project ECHO Ontario, Toronto, Ontario, Canada; ^b^Dalhousie University, Halifax, Nova Scotia, Canada; ^c^President Canadian Pain Association of Canada, Toronto, Ontario, Canada

**CONTACT** Ruth Dubin office@doctorruthdubin.ca


^*^Symposium Chair


© 2017 Ruth Dubin, Maureen Allen, and Terry Bremner. Published with license by Taylor & Francis.

This is an Open Access article distributed under the terms of the Creative Commons Attribution License (http://creativecommons.org/licenses/by/3.0/), which permits unrestricted use, distribution, and reproduction in any medium, provided the original work is properly cited. The moral rights of the named author(s) have been asserted.

Canada is in the grips of an opioid overdose crisis which has multiple root causes, including inadequately trained healthcare providers, long wait times to access pain clinics and specialists plus other systemic barriers to safe and effective pain management. Regulatory colleges, professional bodies and health ministries are now working to mitigate the tragic consequences of poor pain management and the over-prescribing of opioid analgesics.

Since Watt-Watson et al’s 2009 article outlined the gulf between pain education for veterinarians versus human HCP’s, a number of Canadian institutions have developed strong educational programs. However other institutions lag behind despite strong advocacy from both patients and professionals.

This presentation will share some successful community and educational interventions as well as some persistent barriers to best-practice pain education and clinical services delivery for Canadians who live with chronic pain and healthcare providers who support them. Audience input into seeking solutions will be encouraged.

## Speaker 1 Abstract Title: *My View from the trenches: Pain Patients and the opioid crisis*

**Speaker 1 Name**: Terry Bremner

**Speaker 1 Affiliation**: President Canadian Pain Association of Canada, Toronto, Ontario, Canada

**Speaker 1 Abstract**: National focus on the “opioid crisis” has ignored the related crises of poorly managed pain in Canada, and inadequately trained healthcare professionals. People living with chronic pain have long described difficulty finding primary care providers to take them into their practices. However, widespread concerns about regulatory colleges and messages like “just say no” to opioids have created even more barriers for pain patients. Some have endured serious medical harms (e.g. strokes, heart attacks, suicides) from being “cut off” from their opiate medications or being tapered too quickly. Mr. Bremner’s presentation will report on these harms, as well as spill-over effects for other suffering populations such as people with opiate addiction or palliative patients worrying at the end of their lives about addiction.

## Speaker 2 Abstract Title: *A community’s response to the opiate + chronic pain epidemic*

**Speaker 2 Name**: Maureen Allen

**Speaker 2 Affiliation**: Dalhousie University, Halifax, Nova Scotia, Canada

**Speaker 2 Abstract**: This presentation will describe an integrated pain and addiction model of care developed in 2011 in a rural emergency department as a response to the region’s opioid crisis. By engaging community partners and healthcare providers, we built support within the community itself. Clinical E.R. staff attended a fundamentals training module which clarified the language around pain and addiction. The program also delivered the basic elements of safe pain management, including risk stratification and harm reduction.

Assessing treatment risks is the norm in other common illnesses such as stroke or heart disease when potentially harmful drugs like thrombolytics are being considered. Shifting both prescriber and patients’ expectations towards understanding the balance between potential harms or benefits of opioids focussed clinical decision-making more on safety and efficacy rather than moral or ethical arguments.

Our local initiative brought compassionate, non-judgemental care based on best practices to an underserved community. It helped to de-stigmatize chronic pain and addiction and offered timely access to comprehensive care.

This talk will describe the program, report on outcomes, and aims to catalyse its dissemination to other jurisdictions.

## Speaker 3 Abstract Title: *The long and winding road to family physician pain training*

**Speaker 3 Name**: Ruth Dubin

**Speaker 3 Affiliation**: Queens University, Kingston, Ontario, Canada and Project ECHO Ontario, Toronto, Ontario, Canada

**Speaker 3 Abstract**: Family Physicians are often the first port of call for Canadians presenting with symptoms of chronic pain.

In 2011, The College of Family Physicians of Canada (CFPC) accepted an application from Canadian Pain Society members to form a chronic pain special interest group (now known as a community of practice in family medicine).

Two of the committee’s goals have been fulfilled, ie to support practicing family physicians via mentorship and education and to be involved in regional, provincial and national strategic planning around chronic pain management.

However, three goals remain to be achieved:

- To explicate the minimum competencies in chronic pain management for all family medicine trainees.
- To ensure that family medicine program directors have the resources to cover the chronic pain competencies.
- To develop chronic pain case scenarios for inclusion in the family medicine examination.

At the CFPC’s 2016 Annual Forum, the Chairs of the addiction and chronic pain committees (Dr. Sharon Cirone, Dr.Ruth Dubin) collected feedback from leaders, program directors, and other attendees on their perceived barriers to providing adequate basic chronic pain and addiction training to family medicine trainees.

This presentation will summarize the qualitative content of this feedback, and will invite session attendees to develop approaches to overcoming barriers and ensuring that all family physicians in Canada have the skills to safely manage the majority of their chronic pain patients within their primary care medical homes.

## Learning Objectives

1. Highlight barriers to safe and accessible healthcare for pain patients due to declaration of the opioid crisis.

2. Understand some advances in pain education for primary care providers as well as persistent barriers

3. Celebrate (and perhaps replicate) an innovative solution for managing both addiction and pain in a community setting.

## Pain control, perception and expression in older individuals

Magali Millecamps^a,*^, Thomas Hadjistavropoulos^b^, and Guillaume Léonard^c^

^a^Alan Edwards Center for Research on Pain, Dentistry Department, McGill University, Montréal, Québec, Canada; ^b^Research Chair in Aging and Health, Director, Centre on Aging and Health, Department of Psychology and Centre on Aging and Health University of Regina, Saskatchewan, Canada; ^c^School of Rehabilitation, Université de Sherbrooke, Research Center on Aging Centre intégré universitaire de santé et de services sociaux de l’Estrie – Centre hospitalier universitaire de Sherbrooke (CIUSSS de l’Estrie-CHUS), Québec, Canada

**CONTACT** Magali Millecamps magali.millecamps@mcgill.ca


^*^Symposiums Chair


© 2017 Magali Millecamps, Thomas Hadjistavropoulos, and Guillaume Léonard. Published with license by Taylor & Francis.

This is an Open Access article distributed under the terms of the Creative Commons Attribution License (http://creativecommons.org/licenses/by/3.0/), which permits unrestricted use, distribution, and reproduction in any medium, provided the original work is properly cited. The moral rights of the named author(s) have been asserted.

One in five Canadians experience chronic pain. Pain prevalence increases with age. Adults over 65 often present with numerous pathological conditions which results in over prescription of medications. This is concerning because older people are also more sensitive to drug interactions and side effects. Moreover, co-morbidities like cognitive decline, associated with aging, often complicate pain assessment and management. While they are among the most likely to suffer from chronic pain, older adults end up being the least likely to receive adequate treatment.

To improve pain management in older populations, it is necessary to improve our understanding of pain experience and physiology in older individuals. Ultimately, a better understanding of pain in geriatric populations will result in the optimization of pain. New therapeutic options, more suitable for the special needs of this growing population, are urgently needed.

After a brief introduction of the clinical problem, our multidisciplinary and translational panel of experts will explore three aspects of this growing field. Thomas Hadjistavropoulos will present the clinical challenges of assessing pain in older adults including those suffering from severe dementia. Magali Millecamps will describe changes in the behavioural expression of acute versus chronic pain in aging mice and investigate the possible underlying supra-spinal mechanisms. Guillaume Léonard will conclude our symposium with a presentation on pain management in older patients focusing on non-pharmacological alternatives.

## Speaker 1 Abstract Title: *Pain communication in older adults: Self-report, nonverbal expressions and clinical challenges*

**Speaker 1 Name**: Thomas Hadjistavropoulos

**Speaker 1 Affiliation**: Research Chair in Aging and Health, Director, Centre on Aging and Health, Department of Psychology and Centre on Aging and Health University of Regina, Saskatchewan, Canada

**Speaker 1 Abstract**: The subjective experience of pain is normally encoded in expressive behavior which, in turn, can be decoded by observers who may be in a position to provide the necessary care and assistance. Examinations of pain behaviour have led to the conclusion that older adults tend to be more stoic and relatively less likely to report their pain than are younger individuals. This finding has been attributed to a variety of causes including the possibility that older adults may consider pain to be a normal part of aging that needs to be endured. Despite less frequent pain reports, experimental evidence from several studies is consistent with reductions in pain tolerance as a function of age. Studies of nonverbal pain expressions have shown that the facial reactions to pain remain relatively preserved in older age groups, including populations with mild cognitive impairment, supporting the importance of nonverbal pain expressions in pain assessment. Nonverbal pain expressions play an even more central role in the case of older individuals who cannot express pain verbally due to severe dementia. Pain in these populations can be assessed effectively using clinically useful standardized observational procedures. Nonetheless, complicating the pain assessment of the older adult, are documented observer biases (e.g., stereotypes) that can lead to inaccurate judgments about an older person’s pain. Recommendations for addressing challenges in the pain assessment of the older individual will be presented.

## Speaker 2 Abstract Title: *Changes in behavioral expressions of acute and chronic pain in aging mice*

**Speaker 2 Name**: Magali Millecamps

**Speaker 2 Affiliation**: Alan Edwards Center for Research on Pain, Dentistry Department, McGill University, Montréal, Québec, Canada

**Speaker 2 Abstract**: Chronic pain prevalence increases with age. Advanced age is also associated with a general slowdown of many biological processes including neurological function, changes in sensory function and cognitive impairment. A better understanding of the pain experience during aging is needed. This presentation will focus on evidence and results from basic science.

In a series of experiments using young (3-month) and old (24-month) male mice, we investigated the impact of aging on sensory, cognitive and affective spontaneous behaviours in different pain contexts. First, we assessed naïve, healthy aging mice and determined the relationship between each behavioural response and advanced age. Our results demonstrated that aging mice develop both cognitive decline and sensory hypersensitivity, which are co-morbid. Second, we investigated the impact of living with chronic neuropathic pain (Partial Sciatic Nerve Ligation, PNL) on behavioural measures. Our results demonstrate that older animals exhibit fewer behavioural signs of ongoing pain than young mice in several assays including pain aversion assessment. To summarize, naïve aging mice are more sensitive to acute painful stimuli than their younger counterparts, but when they live daily with a chronic pain condition, they display reduced pain behaviour. Given the apparent discrepancy in pain experience between aged naïve (hypersensitive) versus aged chronic neuropathy (hypo-sensitive) mice, we investigated supra-spinal plasticity. We measured levels of different key neurotransmitters and neuromodulators. Our results suggest that supra-spinal plasticity taking place during pain persistence has a different biochemical signature in young vs. old animals.

## Speaker 3 Abstract Title: *Neurostimulation as an alternative approach to pharmacological treatment for alleviating pain in older adults*

**Speaker 3 Name**: Guillaume Léonard

**Speaker 3 Affiliation**: School of Rehabilitation, Université de Sherbrooke, Research Center on Aging Centre intégré universitaire de santé et de services sociaux de l’Estrie – Centre hospitalier universitaire de Sherbrooke (CIUSSS de l’Estrie-CHUS), Québec, Canada

**Speaker 3 Abstract**: Chronic pain is highly prevalent in older adults, who are often heavily medicated and prone to medication side effects. The American Geriatrics Society recommends combining both pharmacological and non-pharmacological treatment options for seniors suffering from persistent pain. This strategy, known as multimodal analgesia, has been shown to optimize patient comfort, while reducing the side effects of medication. Both of these advantages are of interest to clinicians working with older persons. This presentation will tackle the potential utility peripheral and transcranial neurostimulation techniques in elderly individuals suffering from chronic pain. Mechanisms of action of these non-pharmacological approaches will be described and discussed in regards to the changes occurring in the central nervous system of older individuals. Efficacy and effectiveness evidence will be presented, with an emphasis placed on studies conducted in older populations. New and emerging neurostimulation treatments, such as non-invasive brain stimulation (NIBS), will be examined, and preliminary evidence regarding the efficacy of transcranial direct current stimulation in older individuals suffering from chronic musculoskeletal pain will be presented. A better knowledge of the mechanisms of action and of the evidence regarding the clinical efficacy of these established and emerging treatments will guide clinicians in their decision-making and pave the way for future research.

## Learning Objectives

1. To familiarize participants with clinical challenges in the assessment of pain in older adults and with ways of overcoming these challenges.

2. To provide evidence of changes in the chronic pain experience during aging and discuss possible underlying mechanisms.

3. To explore new non-pharmacological pain management avenues to help older patients manage chronic pain.

## The evolutionary biology of pain

Jeffrey S. Mogil^a,*^, Amanda C. de C. Williams^b^, and Edgar T. Walters^c^

^a^E.P. Taylor Professor of Pain Studies, Canada Research Chair in Genetics of Pain (Tier I), Dept. of Psychology, McGill University, Director, Alan Edwards Centre for Research on Pain, Montreal, QC, Canada; ^b^Reader, Research Department of Clinical, Health and Educational Psychology, University College London, London, United Kingdom; ^c^Fondren Chair in Cellular Signaling, Ray A. and Robert L. Kroc Faculty Fellow, Professor of Integrative Biology and Pharmacology, McGovern Medical School at UTHealth, Houston, TX, USA

**CONTACT** Jeffrey S. Mogil jeffrey.mogil@mcgill.ca


^*^Symposium Chair


© 2017 Jeffrey Mogil, Amanda C. de C. Williams, and Edgar T. Walters. Published with license by Taylor & Francis

This is an Open Access article distributed under the terms of the Creative Commons Attribution License (http://creativecommons.org/licenses/by/3.0/), which permits unrestricted use, distribution, and reproduction in any medium, provided the original work is properly cited. The moral rights of the named author(s) have been asserted.

Evolutionary theory has proven to be a useful way to understand biological phenomena, but has almost never been applied to pain. Even the simplest of questions remained unanswered: What is the purpose of pain, including chronic pain? Do animals approach or avoid pain in others? How similar are mechanisms of pain-like states in phylogenetically diverse animals? The presenters will discuss theory and experimental data, collected in animals and humans, bearing upon these issues. Amanda Williams will discuss theoretical issues and introduce relevant evolutionary concepts. Edgar (Terry) Walters will discuss the evolution of nociceptive sensitization mechanisms, presenting related observations from Aplysia, squid, insects, fish, and rodents. Jeffrey Mogil will discuss the surprising evolutionary conservation of social modulation of pain, describing parallel experiments performed in rodents and humans.

## Speaker 1 Abstract Title*: Evolution pain behaviour, and the persistence of pain*

**Speaker 1 Name**: Amanda C. de C. Williams

**Speaker 1 Affiliation**: Reader, Research Department of Clinical, Health and Educational Psychology, University College London, London, United Kingdom

**Speaker 1 Abstract**: Acute pain is generally agreed to be an essential survival mechanism across vertebrates and invertebrates, producing physiological and behavioural changes that promote healing and recovery. There are divergent theories concerning chronic pain: that it has no function; that it results from a mismatch between our evolutionary environment and our current conditions; or that chronic pain evolved to elicit help. We know almost nothing about pain over human evolution, except that some of our ancestors survived major injury, so help from others appears likely. Observation of nonhuman primates and from other mammals in pain identifies common behaviours with clear functions in relation to recovery from injury. This contrasts strongly with accounts of the same behaviours in humans that refer to operant reinforcement or to mood disorders such as anxiety and depression. Understanding the function (if any) of chronic pain and of related behaviours may offer new interventions to reduce the persistence of pain.

## Speaker 2 Abstract Title: *Evolutionary insights into mechanisms that drive persistent pain*

**Speaker 2 Name**: Edgar T. Walters

**Speaker 2 Affiliation**: Fondren Chair in Cellular Signaling, Professor of Integrative Biology and Pharmacology, McGovern Medical School at UTHealth, Houston, TX, USA

**Speaker 2 Abstract**: Persistent pain is notoriously difficult to manage. While considered maladaptive clinically, a broader biological perspective suggests that the resistance of persistent pain to treatment might be a consequence of strong evolutionary selection pressures for redundant mechanisms to ensure that an animal remains hypervigilant while slowly recovering from severe injury, and that it maintains nociceptive hypersensitivity in an injured region even if that region loses effective sensory innervation. Such selection pressures are likely to have shaped ancient mechanisms of nociceptive sensitization that may have been widely conserved. This possibility is supported by striking similarities across animal phyla in physiological and molecular mechanisms that produce long-lasting hypersensitivity of nociceptive pathways after injury. The most ancient mechanisms may be expressed in primary sensory neurons specialized to detect peripheral injury (nociceptors), which probably evolved earlier than complex central networks. Indeed, persistent electrophysiological and morphological alterations in nociceptors have been found that are similar after peripheral injury in several invertebrate species, rodents, and humans, suggesting very early origins and extensive conservation. Similar alterations in the cell bodies and peripheral terminals of nociceptors are also triggered in mammals by central (spinal cord) injury. These and other observations suggest that evolution has endowed nociceptors with ancient mechanisms that detect various combinations of signals associated with severe bodily injury. These fundamental mechanisms may switch nociceptors into a persistent hyperfunctional state that, in mammals, can drive chronic pain even when a nociceptor’s peripheral branches have been disconnected by injury or when peripheral sensitization mechanisms are inhibited therapeutically.

## Speaker 3 Abstract Title: *Mice are people too: Social modulation of and by pain in laboratory rodents and humans*

**Speaker 3 Name**: Jeffrey S. Mogil

**Speaker 3 Affiliation**: E.P. Taylor Professor of Pain Studies, Canada Research Chair in Genetics of Pain (Tier I), Dept. of Psychology, McGill University, Director, Alan Edwards Centre for Research on Pain, 1205 Dr. Penfield Ave., Montreal, QC H3A 1B1, Canada

**Speaker 3 Abstract**: It is a standard assumption of the biopsychosocial model that whereas biological factors are better studied in animal models, psychological and especially social factors can only be studied in human beings. Furthermore, many believe phenomena such as empathy and prosocial behaviours to be the sole province of humans. However, the evolutionary antecedents of such phenomena are starting to be demonstrated in non-human animals, and even in rodents. I will discuss recent experiments in my lab and others’ showing the effect of social communication on pain behaviour, and the effect of pain on social interactions. The surprisingly successful translation of findings from mice to undergraduates suggests that pain modulation is highly conserved across mammalian species.

## Learning Objectives

1. To understand the potential utility of evolutionary theory as applied to pain-related phenomena

2. To consider evidence for widespread, adaptive, pain-like states in the animal kingdom

3. To appreciate new evidence related to social modulation of pain in animals and humans

## Procedure pain management for chronic and critically ill pediatric and adult patients: Identification, innovation, and evidence synthesis

Jennifer Stinson^a,*^, Kathryn Birnie^a^, and Craig Dale^b^

^a^Hospital for Sick Children / University of Toronto, Toronto, Ontario, Canada; ^b^University of Toronto / Sunnybrook Health Sciences Centre, Toronto, Ontario, Canada

**CONTACT** Dr. Kathryn Birnie kbirnie@dal.ca


^*^Symposium Chair


© 2017 Jennifer Stinson, Kathryn Birnie, and Craig Dale. Published with license by Taylor & Francis.

This is an Open Access article distributed under the terms of the Creative Commons Attribution License (http://creativecommons.org/licenses/by/3.0/), which permits unrestricted use, distribution, and reproduction in any medium, provided the original work is properly cited. The moral rights of the named author(s) have been asserted.

Medical procedures are an incredibly common source of pain and distress, particularly for individuals requiring frequent and/or repeated procedures for assessment or treatment. Despite the frequent occurrence of medical procedures for the chronic or critically ill patient, strategies to manage procedural pain are often underutilized and reports of significant pain remain high for pediatric and adult patients alike (e.g., Siffleet et al., 2007; Stevens et al., 2011). Furthermore, inadequate management of procedure-related pain and distress can significantly negatively impact subsequent procedures and patient’s recovery. The goal of this symposium is to highlight emerging innovative research in the area of procedure pain assessment and management. In particular, it focuses on the need for further research and clinical attention regarding procedure pain management for chronically and critically ill patients with whom more limited work has been done to date. The workshop will: (1) review the evidence for psychological/behavioural management of procedure-related pain and distress from early childhood to adulthood, with particular attention to the evidence derived from chronically or critically ill patient populations; (2) present evidence for the application of new technology (MEDi, a humanoid robot) for procedure management in children with cancer; and (3) discuss new investigations of the prevalence of pain behaviours during oral care procedures for mechanically ventilated adults in the intensive care unit.

## Speaker 1 Abstract Title: *Evidence of what and for whom? Psychological interventions for procedure related pain and distress in children, adolescents, and adults*

**Speaker 1 Name:** Dr. Kathryn Birnie

**Speaker 1 Affiliation:** Hospital for Sick Children / University of Toronto, Toronto, Ontario, Canada

**Speaker 1 Abstract:** Psychological strategies are one part of a multi-pronged approach used to mitigate procedural pain and distress, in addition to pharmacological, physical, and procedural interventions. Psychological interventions comprise cognitive and/or behavioural strategies and have demonstrated efficacy in a variety of settings and populations. However, not all studied interventions are efficacious and evidence is not available for all patient populations. Furthermore, some psychological interventions are time-consuming and require additional effort or ‘man-power’ to implement effectively. Given this, it is critical to identify evidence-based psychological interventions with application to specific care settings or specific patient populations. This presentation will review evidence for the psychological management of pain and distress from medical procedures for children, adolescents, and adults. Findings will be shared from a number of recent systematic reviews and meta-analyses with particular focus on evidence derived from chronically and/or critically ill patients, such as children with cancer and adults in intensive care. Data will be presented from a newly updated Cochrane review of published randomized controlled trials examining psychological interventions for needle-related procedures in children and adolescents aged 2-19 years. Database searches were conducted in September of 2016 with 25 new trials identified since the last published review in 2013. This presentation will inform clinicians’ selection of effective psychological interventions for their setting.

## Speaker 2 Abstract Title: *MEDi-Port: Using a humanoid robot to reduce procedural pain and distress in children with cancer: A pilot randomized controlled trial*

**Speaker 2 Name:** Dr. Jennifer Stinson

**Speaker 2 Affiliation:** Hospital for Sick Children /University of Toronto, Toronto, Ontario, Canada

**Speaker 2 Abstract:** Pain is a frequent and significant problem for children with cancer. Needle procedures are reported by children to be the most distressing experience caused by cancer and its treatment. A specific program has been developed for MEDi-Port, a small interactive robot, to decrease pain and distress in children with cancer undergoing subcutaneous port (SCP) access. The aim of this pilot randomized controlled trial was to compare the MEDi-Port intervention to an active control for reducing pain and distress during SCP. Cancer patients (n=40) aged 4-9 years old (60% boys) rated pain and distress prior to their SCP access. During the procedure, MEDi performed either an SCP-specific guided intervention or dancing, singing and storytelling as a control. Patients, parents, and nurses rated the child’s post-procedure pain, distress, and satisfaction. Parents reported high satisfaction – 62% thought MEDi was very helpful for reducing their child’s pain. Children from both the intervention and control groups reported a mean pain reduction of 1.87 points on an 11-point scale as compared to their last SCP access, although there were no significant differences in pain or distress ratings by children, parents, or nurses observed between MEDi-Port and MEDi active control groups. In addition, 67% of children enjoyed the presence of MEDi very much. Technical problems accounted for most of the low satisfaction and effectiveness ratings. Next steps include conducting a definitive randomized controlled trial and implementing MEDi in the oncology setting.

## Speaker 3 Abstract Title: *Under-recognized and undertreated: Procedural oral pain in the adult intensive care unit*

**Speaker 3 Name:** Dr. Craig Dale

**Speaker 3 Affiliation:** University of Toronto /Sunnybrook Health Sciences Centre, Toronto, Ontario, Canada

**Speaker 3 Abstract:** Routine procedures are an important source of pain for adult intensive care unit (ICU) patients. Emerging research demonstrates procedural oral pain is an under-recognized and undertreated dimension of this problem. Oral hygiene comprises seemingly innocuous procedures performed repeatedly over the course of a typical day in ICU. However, these care processes may elicit pain responses in the context of altered oral tissue states common in critical illness. Current guidelines offer little guidance for the assessment and treatment of procedure-related oral pain. One reason for this omission is the lack of empirical data regarding the experiences and responses of non-verbal mechanically ventilated patients to common oral procedures. In order to fill this knowledge gap, we undertook a prospective multicenter observational study of 428 adults (≥ 18 years old) orally intubated for ≥ 48 hours in 3 academic and 1 community ICU to assess prevalence of possible pain behaviours during common oral procedures (oral swabbing, toothbrushing, and suctioning). Patient behaviours during routine oral interventions (suctioning, swabbing and toothbrushing) included coughing/gagging (59.6%), mouth closing (49.3%), biting (44.6%), localizing (27.1%), turning the head side-to-side (23.4%), sitting up (14.5%) and hitting (8.2%). In qualitative follow-up interviews, ICU survivors recalled most oral hygiene procedures and confirmed untreated oral discomfort (82%) and pain (52%). As assessment is the cornerstone of pain management, new research to validate a behavioral tool to detect procedural oral pain is warranted.

## Learning Objectives

1. Identify evidence-based psychological interventions for procedure pain management from childhood through adulthood, including direct evidence for chronically or critically ill patients.

2. Describe the application, benefits, and challenges of using a humanoid robot (MEDi-Port) for pain and distress in children with cancer undergoing subcutaneous port access.

3. Gain new insight into procedural oral pain in critical illness including patient behaviours and recollections.

## Biomarkers for chronic pain

Javeria Ali Hashmi^a,*^, Karen Davis 0000-0003-1879-0090 ^b^, and Jason J. McDougall^a^

^a^Department of Anesthesia, Pain Management & Perioperative Medicine, Dalhousie University, Halifax, Nova Scotia, Canada; ^b^Department of Surgery and Institute of Medical Science, University of Toronto, Head, Division of Brain, Imaging and Behaviour-Systems Neuroscience, Krembil Research Institute, Toronto Western Hospital, University Health Network, Toronto, Ontario, Canada

**CONTACT** Javeria Ali Hashmi javeria.hashmi@dal.ca


^*^Symposium Chair


© 2017 Javeria Ali Hashmi, Karen Davis, and Jason J. McDougall. Published with license by Taylor & Francis

This is an Open Access article distributed under the terms of the Creative Commons Attribution License (http://creativecommons.org/licenses/by/3.0/), which permits unrestricted use, distribution, and reproduction in any medium, provided the original work is properly cited. The moral rights of the named author(s) have been asserted.

**Symposium Abstract** Chronic pain is associated with changes in nerve morphology, signaling properties from peripheral nerve terminals to the brain, and/or changes within the brain itself. However, the experience and report of pain are subjective, can be intransitive in nature, and do not necessarily correlate with underlying pathologies. As a result, there is no objective diagnostic test to complement the subjective assessment of chronic pain. The fact that there are no objective biomarkers for chronic pain has cast a pall on how to diagnose, understand and treat the often incendiary reports of severe pain in such a large population of people. Objective information on the neurobiological causes that mediate chronic pain could dramatically change the current outlook for treatment of chronic pain and help to validate the biological origin of chronic pain.

In this symposium, the speakers will outline current efforts to delineate biomarkers of chronic pain by using different research approaches and techniques. Dr. Karen Davis will discuss new directions for developing objective biomarkers of treatment outcomes from brain imaging for 1) trigeminal neuralgia, 2) ankylosing spondylitis, and 3) peripheral nerve injuries. Dr. Jason McDougall will share novel information on neural biomarkers that specifically signal the “chronic pain” aspects of tissue damage associated with knee osteoarthritis. Dr. Hashmi’s talk will underscore the potential uses of neural biomarkers for predicting treatment outcomes for chronic pain. Her talk will highlight the necessity, significance, potential impact, and models for using neural biomarkers to implement precision medicine in chronic pain therapeutics.

## Speaker 1 Abstract Title: *Neuroimaging of chronic pain treatment effects*

**Speaker 1 Name**: Karen Davis

**Speaker 1 Affiliation**: Professor, Department of Surgery and Institute of Medical Science, University of Toronto, Head, Division of Brain, Imaging and Behaviour-Systems Neuroscience, Krembil Research Institute, Toronto Western Hospital, University Health Network, Toronto, Ontario, Canada

**Speaker 1 Abstract**: For over 20 years, brain imaging studies have revealed that patients with chronic pain have both structural and functional brain abnormalities in areas such as the insula, somatosensory and cingulate cortex, and the prefrontal cortex. There is also evidence of structural abnormalities in the trigeminal nerves of patients with orofacial pain conditions such as trigeminal neuralgia and temporomandibular disorder. More recent studies have sought to determine how treatment impacts these abnormalities and whether there are specific pre-treatment baseline indicators of treatment outcome that can be used as prognosticators. In this talk, I will present data that demonstrate the capability of the brain and trigeminal nerves to undergo treatment-related plasticity that normalize structure and function from studies in patients with chronic pain due to 1) trigeminal neuralgia, 2) ankylosing spondylitis, and 3) peripheral nerve injuries. The most striking findings from these studies indicate that brain abnormalities are impacted by pain treatments and that normalization of abnormalities in the insula and trigeminal nerves are related to the degree of pain relief. Furthermore, I will present data of potential prognosticators of treatment outcome. These findings provide early evidence that neuroimaging has the potential to contribute personalized pain management.

## Speaker 2 Abstract Title: *Discovery of a novel biomarker for neuropathic pain in ostoearthritis*

**Speaker 2 Name**: Jason J. McDougall

**Speaker 2 Affiliation**: Professor Department of Pharmacology, Department of Anesthesia, Pain Management & Perioperative Medicine, Dalhousie University, Halifax, Nova Scotia, Canada

**Speaker 2 Abstract**: In addition to inflammatory and nociceptive pain, a subset of osteoarthritis (OA) patients are believed to suffer from neuropathic pain. This presentation will provide recent evidence showing that OA joint nerves become damaged during OA progression and spontaneously fire indicative of neuropathic pain. OA patients describe neuropathic-pain like symptoms and score highly on PainDETECT questionnaires. Finally drugs that are used to treat neuropathic pain (e.g. gabapentinoids, duloxetine) are effective in a subpopulation of OA patients. The cause of this peripheral neuropathy and the development of neuropathic pain in OA patients is unknown. The lipid mediator lysophosphatidic acid (LPA) has been described as a signature molecule in the destruction of afferent nerves and is a major contributor to neuropathic pain. I will present experimental evidence showing that LPA is present in the synovial fluid of OA patients and that its levels correlate with disease severity. In animal experiments, I will show that an intra-articular injection of LPA in rats causes joint peripheral neuropathy, joint damage and pain. Finally, treatment of OA rats with an LPA antagonist reduced neuropathic pain-like behaviour and sensory neuropathy. Thus, LPA provides a missing link between OA and joint neuropathic pain.

## Speaker 3 Abstract Title: *Data analytics for implementing precision medicine in chronic pain therapeutics*

**Speaker 3 Name**: Javeria Ali Hashmi

**Speaker 3 Affiliation**: Canada Research Chair Tier II (Pain), Assistant Professor, Department of Anesthesia, Pain Management & Perioperative Medicine, Dalhousie University, Halifax, Nova Scotia, Canada

**Speaker 3 Abstract**: The advent of new imaging methods and data modeling techniques has revolutionized our capacity to understand and utilize brain signals. With these techniques, the dynamic interactions between the complex elements of the brain can be used to effectively decode the neurological substrates of pain perception and modulation. The task to discover how intricately connected brain networks process psychological cues and interpret them into analgesia is an important scientific challenge that can leeway into novel clinical tools and improved pain therapeutics. This talk will present evidence that brain network properties can be used to predict the intrinsically mediated aspects of treatment outcome such as placebo response. The talk will also highlight how brain imaging also provides clues into maladaptive pain processing associated with chronic pain. Neuroimaging methods combined with current approaches for data modelling offer a new framework for implementing Big Data methods such as graph theory and machine learning for building biomarkers for treating chronic pain. The speaker/chair will define and discuss how these findings can be implemented within a precision medicine framework and will also discuss caveats and potential pitfalls of current biomarker research that need to be addressed when developing precision medicine approaches for conditions such as chronic pain that have a neuropsychiatric dimension.

## Learning Objectives

1. To share novel evidence for neural involvement in chronic pain in the peripheral and central nervous systems through research in humans and animals

2. Project how neurobiological findings can serve to more accurately diagnose, understand and treat chronic pain and improve the future of chronic pain diagnostics and clinical pain management

3. Discuss caveats and potential pitfalls of existing precision medicine models

## Protective responses in chronic pain and how they become maladaptive

Tasha R. Stanton^a,*^, Geoffrey P. Bostick^b^, and Ann Meulders^c^

^a^University of South Australia, Adelaide & Neuroscience Research Australia, Sydney, Australia; ^b^University of Alberta, Edmonton, Alberta, Canada; ^c^KU Leuven, Leuven, Belgium and Maastricht University, Maastricht, The Netherlands

**CONTACT** Tasha Stanton tasha.stanton@unisa.edu.au


^*^Symposium Chair


© 2017 Tasha R. Stanton, Geoff P. Bostick, and Ann Meulders. Published with license by Taylor & Francis.

This is an Open Access article distributed under the terms of the Creative Commons Attribution License (http://creativecommons.org/licenses/by/3.0/), which permits unrestricted use, distribution, and reproduction in any medium, provided the original work is properly cited. The moral rights of the named author(s) have been asserted.

This symposium aims to explore the complexity of protective responses in chronic pain. Learning to predict danger is critical to human survival; it allows us to both anticipate and avoid harm. Perhaps unsurprisingly then, people in pain are often very protective of their painful body part – they limit movement and avoid activities that are perceived as being threatening or that may provoke pain. These protective responses are adaptive in the acute pain stage and promote healing and recovery, yet they might paradoxically aggravate the situation when pain becomes chronic and no longer signals bodily threat. This symposium will specifically focus on how initially adaptive responses may become maladaptive in chronic pain and also how a similar response to pain may be adaptive in one context, but maladaptive in another. Recently, several innovative lines of research on the role of bodily perception, expectation, and learning in protective responses to pain have emerged. Dr Tasha Stanton will discuss new findings in chronic low back pain suggesting that bodily feelings of stiffness may represent a maladaptive protective response against movement. Dr Geoff Bostick will present work showing that in chronic pain, the same expectation of outcome can be adaptive in one context, but maladaptive in another: that is, those with failed treatments update their expectations regarding outcome, allowing for reductions in maladaptive responses such as pain catastrophizing. Last, Dr Ann Meulders will present new data on associative learning in pain, specifically the role of maladaptive generalisation and the lack of selective learning; features which may well underlie the transition between maladaptive and adaptive responses and/or maintain chronic pain problems.

## Speaker 1 Abstract Title: *Bodily feelings of stiffness – a maladaptive response to protect against movement?*

**Speaker 1 Name**: Tasha Stanton

**Speaker 1 Affiliation**: University of South Australia, Adelaide & Neuroscience Research Australia, Sydney, Australia

**Speaker 1 Abstract**: Bodily feelings, such as pain, are often protective in that they allow us to safeguard our body from further damage by avoiding provoking activities. We often assume that bodily feelings reflect the biological state of our body tissues. Yet, there is growing body of evidence to suggest that our bodily feelings are a result of complex multisensory processes, driven by behaviorally relevant outcomes, the aim of which is to maintain homeostatic regulation of the body. One feeling that is almost universally experienced, which impacts on our daily lives, but which remains relatively unstudied, is feeling stiff. For example, feeling stiffness in one’s back is unanimously attributed to actually having a stiff back. But is this true? In this presentation, I will describe a series of experiments, the results of which collectively compel us to rethink what it means to feel stiff, what determines how stiff we feel and what ecological value feeling stiff might have. I will discuss the idea that a feeling of stiffness is a multisensory perceptual illusion that can act as a maladaptive protective response against movement.

## Speaker 2 Abstract Title: *Pain expectation in people with chronic pain: a shift from maladaptive to adaptive responses?*

**Speaker 2 Name**: Geoff Bostick

**Speaker 2 Affiliation**: University of Alberta, Edmonton, Alberta, Canada

**Speaker 2 Abstract**: Expectations of future outcomes have been shown to predict who goes on to develop chronic pain. Much of the literature supports a self-fulfilment perspective. That is, those with positive expectations tend to have more positive outcomes while those with negative expectations tend to have more negative outcomes. However, in cases of chronic refractory pain, positive expectations for pain reduction may be maladaptive as they could impede progress in rehabilitation approaches to improving function and quality of life despite unresolved pain. These initially adaptive expectations may drive maladaptive protective responses, such as developing high levels of pain catastrophizing or distress when expectations for pain reduction are not met, which then can limit function. Thus, the relationship between expectation, pain and maladaptive responses is not clear-cut, and the self-fulfilment perspective may be too simplistic. Further compounding the challenge is the similarity of constructs such as positive expectations, hope and optimism. I will present new research that suggests that expectation of outcome may become adaptive in some chronic pain samples, in a particular context. Namely that altered expectations, that more accurately reflect outcome, may reduce pain catastrophizing. In addition, I will present data to help illustrate the challenges in unravelling the adaptive role of optimism and the potential maladaptive role of unrealistic expectations.

## Speaker 3 Abstract Title: *Persistent overgeneralization and lack of selective learning in chronic pain*

**Speaker 3 Name**: Ann Meulders

**Speaker 3 Affiliation**: KU Leuven, Leuven, Belgium and Maastricht University, Maastricht, The Netherlands

**Speaker 3 Abstract**: Anomalies in fear learning, such as the failure to inhibit fear, lead to sustained anxiety, which in turn may augment pain. Stimulus generalization usually is adaptive as it enables individuals to extrapolate the predictive value of one stimulus to similar stimuli without having to learn anew. Yet, when fear spreads in an unbridled way to novel technically safe stimuli, generalization becomes maladaptive and may lead to dysfunctional avoidance behaviors culminate in severe pain disability. Contingency learning, in particular the formation of pain expectancy beliefs, underpins the development of conditioned fear and avoidance, yet equally important is the formation of safety beliefs. I will present a series of experiments showing fear overgeneralization and a lack of selective contingency learning in chronic pain patients. We showed that (1) pain-related fear spreads towards novel movements sharing proprioceptive features with the original pain-associated movement in healthy participants, but in a non-differential way in fibromyalgia patients. In particular, patients’ fear provoked by novel movements did not depend on the similarity with the original pain-eliciting stimulus; instead they overgeneralized their fear to all novel movements. (2) These protective responses persisted despite corrective feedback in patients but not in healthy controls (extinction). (3) In a blocking procedure, in which one event (A+) causes pain and another event occurring together with the pain-eliciting event (AX+) also causes pain, healthy participants typically learn that X is redundant, because A causes the pain, leading to blocking of conditioned responding to X. Patients however failed to show selective learning.

## Learning Objectives

1. To understand how conscious bodily feelings themselves may be maladaptive in chronic pain.

2. To appreciate how cognitively-driven protective responses can become adaptive in the chronic pain patient.

3. To better understand how selective learning underlies maladaptive protective responses and how the lack thereof may play a role in pain chronicity.

## Mindfulness-Based Interventions for chronic pain: Experience and evidence

Ted Robinson^a,*^, Denise Paneduro^b,c^, Jaisa Sulit^c^, and Kareena Gurbaxani^b,c^

^a^University of Toronto Faculty of Medicine, Department of Family & Community Medicine; Wasser Pain Management Center, Mount Sinai Hospital; Bridgepoint Hospital, Toronto, Ontario, Canada; ^b^Wasser Pain Management Center, Mount Sinai Hospital; ^c^York University, Toronto, Ontario, Canada; University of British Columbia, Faculty of medicine, Department of Occupational Science and Occupational Therapy, Vancouver, British Columbia, Canada

**CONTACT** Ted Robinson trob@sympatico.ca


^*^Symposium Chair


© 2017 Ted Robinson, Denise Paneduro, Jaisa Sulit, and Kareena Gurbaxani. Published with license by Taylor & Francis.

This is an Open Access article distributed under the terms of the Creative Commons Attribution License (http://creativecommons.org/licenses/by/3.0/), which permits unrestricted use, distribution, and reproduction in any medium, provided the original work is properly cited. The moral rights of the named author(s) have been asserted.

Chronic pain is a multidimensional life experience. Traditional medical approaches are often limited to treating physical aspects of pain. Biopsychosocial theorectical models of pain (Gatchel, Peng, Peters, Fuchs, & Turk, 2007) and evaluations of mindfulness-based interventions suggest that pharmacological management of chronic pain is inadequate alone, and is most beneficial when combined with more holistic approaches. Mindfulness practices provide the patient with effective self-management strategies for living with the adversity of chronic pain. In this symposium we will review the history of Mindfuness-Based Stress Reduction (MBSR) programs for chronic pain, examine the evidence for their benefit, explore the effectiveness of Mindfulness from the patient’s perspective, and look at future modifications of the basic MBSR program, which may enhance its benefit. This symposium will also provide participants with an opportunity to experience some introductory Mindfulness practices.

## Speaker 1 Abstract Title: *Evidence for effectiveness of MBSR for chronic pain*

**Speaker 1 Name**: Denise Paneduro

**Speaker 1 Affiliation**: Wasser Pain Management Center, Mount Sinai Hospital; York University, Toronto, Ontario, Canada

**Speaker 1 Abstract**: MBSR has been shown to be beneficial for chronic pain patients for over 35 years. From early studies in the 1980’s at the Center for Mindfulness in Medicine, Health Care and Society at UMass Medical Center to a randomised waitlist controlled trial of the MBSR program for chronic pain at the Wasser Pain Management Center, the evidence continues to document improved health. Dr. Denise Paneduro will review this evidence, including her dissertation research conducted at the Wasser. This study is the first randomised controlled trial to evaluate the impact of MBSR on change blindness, a measure of attention, and to track changes in mindfulness and pain acceptance over time and evaluate their predictive role in improving attention and health. Outcomes assessed included change blindness, pain severity, pain disability, depression, anxiety, stress, mindfulness and pain acceptance. Linear regression analyses using changes scores of the outcomes revealed significantly greater reductions from pre-to-post treatment in the MBSR group compared to the wait-list control group in depression and stress and increases in mindfulness. Multiple linear regression analyses demonstrated that increases in mindfulness significantly predicted decreases in depression and stress, and increases in pain acceptance significantly predicted decreases in pain disability. Overall, findings suggest that MBSR interventions can increase capacity for mindfulness skills among individuals with chronic pain and can serve as an adaptive coping resource for individuals to draw from when faced with stressful and/or painful experiences. Mindfulness-based approaches should be integrated in pain clinics to facilitate patient recovery via reduced emotional distress.

## Speaker 2 Abstract Title: *Mindfulness from inside and Out: Experiences of a chronic pain patient and mindfulness teacher*

**Speaker 2 Name**: Jaisa Sulit

**Speaker 2 Affiliation**: University of British Columbia, Faculty of medicine, Department of Occupational Science and Occupational Therapy, Vancouver, British Columbia, Canada

**Speaker 2 Abstract**: Jaisa Sulit is an Occupational Therapist who sustained a partial spinal cord injury six years ago in a motor vehicle accident. She continues to live with chronic pain and the limitations caused by her injury. Jaisa will discuss her personal experience with mindfulness and its contribution to both her continued rehabilitation and her ability to cope with chronic pain. As a result of the benefits that she experienced personally through mindfulness practice, Jaisa trained at the Center for mindfulness in Medicine, Health Care and Society at UMass Medical Center and has become a qualified MBSR teacher. She will discuss how she has incorporated Mindfulness in her practice of Occupational Therapy - both with individuals and with groups of chronic pain patients.

Jaisa will lead a short mindfulness practice in order to give participants an opportunity to explore mindfulness in an experiential way.

## Speaker 3 Abstract Title: *Mindfulness-Based Cognitive Therapy (MBCT) for chronic pain - a pilot project*

**Speaker 3 Name**: Ted Robinson

**Speaker 3 Affiliation**: University of Toronto Faculty of Medicine, Department of Family & Community Medicine; Wasser Pain Management Center, Mount Sinai Hospital; Bridgepoint Hospital, Toronto, Ontario, Canada

**Speaker 3 Abstract**: Both MBSR and Cognitive-Behavioural Therapy (CBT) have been shown to be effective for improving life among individuals suffering from chronic pain. MBCT was developed initially for relapse prevention in patients with recurrent episodes of depression. Elements of the traditional MBSR and CBT programs currently offered at the Wasser Pain Mangement Center for more than six years were incorporated into a newly developed MBCT curriculum, specifically targeted for chronic pain and introduced at Mount Sinai and Bridgepoint Hospitals in 2016. We hypothesized that, given the reported benefits of MBCT for individuals with depression and the co-occurring high rates of depression among individuals with chronic pain, this new MBCT program might provide enhance benefit through the combination of Mindfulness and CBT self-management strategies. Dr. Ted Robinson will describe the development of the curriculum, its implementation with two pilot groups, and report the pre-to-post-test results of these two groups.

## Learning Objectives

1. Participants will be informed about the evidence that mindfulness-based practices provide chronic pain patients with beneficial coping strategies for living with chronic pain.

2. Participants will have an opportunity to explore mindfulness experientially, and to hear directly from a chronic pain patient about its benefits.

3. Participants will learn about a potentially more powerful new program (MBCT for chronic pain) which incorporates strategies from two programs (MBSR and CBT) both known to be beneficial in chronic pain patients.

## Prescribing movement as medicine for people in pain

Timothy Wideman^a,*^, Neil Pearson^b^, Arthur Woznowski-Vu^a^, and Jordan Miller^c^

^a^McGill University's School of Physical and Occupational Therapy, Montreal, Quebec, Canada; ^b^Department of Physical Therapy, University of British Columbia, Vancouver, British Columbia, Canada; ^c^School of Rehabilitation Therapy, Queen’s University, Kingston, Ontario, Canada

**CONTACT** Timothy Wideman timothy.wideman@mcgill.ca


^*^Symposium Chair


© 2017 Timothy Wideman, Neil Pearson, Arthur Woznowski-Vu, and Jordan Miller. Published with license by Taylor & Francis.

This is an Open Access article distributed under the terms of the Creative Commons Attribution License (http://creativecommons.org/licenses/by/3.0/), which permits unrestricted use, distribution, and reproduction in any medium, provided the original work is properly cited. The moral rights of the named author(s) have been asserted.

The overall aim of this symposium is to describe how prescribing movement and exercise as medicine play a vital role in pain management. Movement and exercise can positively impact the quality of life of an individual with pain by improving function and reducing disability, by providing exercise-induced analgesia, and by reducing unhelpful pain beliefs. The first presentation will describe the analgesic benefits of prescribing exercise as medicine as well as describing how movement can provide a valuable learning opportunity to address unhelpful pain beliefs. The second presentation will introduce participants to the concept of sensitivity to physical activity, which may explain some of the variability in responses to movement and exercise focused interventions. Sensitivity to physical activity measures are associated with increased disability, dysfunctional pain modulation, and negative pain cognitions. These measures can serve as valuable clinical tools by identifying people who are more likely to experience increases in pain following physical activity. This presentation will introduce evidence for the assessment of sensitivity to physical activity in practice and will describe potential implications for tailored approaches to treatment based on sensitivity to physical activity. The final presentation will describe how tailored exercises have been incorporated in a self-management program to improve functional outcomes in a primary health care setting. This example of tailoring exercises to the participant will build on the previous presentations by providing a practical approach to graded activity and exercise for people who are more sensitive to physical activity.

## Speaker 1 Abstract Title: *Using exercise interventions to provide analgesic effects and reduce unhelpful pain beliefs*

**Speaker 1 Name**: Neil Pearson

**Speaker 1 Affiliation**: Department of Physical Therapy, University of British Columbia, Vancouver, British Columbia, Canada

**Speaker 1 Abstract:** Exercise interventions can increase an individual’s functional abilities and reduce pain through exercise induced analgesia. These effects of exercise are well established and understood in the pain care community; however, the use of movement and exercise as educational interventions provides additional opportunities to improve health outcomes with movement and exercise interventions.

Negative patient pain beliefs are significant barriers to positive clinical outcomes. Pain science education is an effective strategy for reducing negative pain beliefs. Yet, for many health professionals, there is little time within the clinical visit to provide the verbal education required to effectively shift beliefs and attitudes to movement and recovery, and for many people in pain didactic learning is not as effective as kinaesthetic or experiential learning. Appropriate movement can be pain relieving, and we now have evidence to inform us on how to teach people to move when painful movement is one of their primary complaints.

The objective of this presentation is to demonstrate how movement and exercise therapies can be used by health care providers to promote exercise induced analgesia, while also providing important educational opportunities to change unhelpful beliefs about pain and movement. Throughout the presentation, examples will be provided to demonstrate how to use brief educational interventions to support our patients’ understanding that movement and exercise are medicine.

## Speaker 2 Abstract Title: *Sensitivity to physical activity as a barrier to participating in physical activity and exercise interventions*

**Speaker 2 Name**: Arthur Woznowski-Vu

**Speaker 2 Affiliation**: School of Physical and Occupational Therapy, McGill University, Montreal, Quebec, Canada

**Speaker 2 Abstract**: Clinical practice guidelines for the treatment of musculoskeletal pain recommend physical activity and exercise interventions. While these interventions aim to reduce pain and associated disability, certain patients may experience an unexpected increase in pain following participation in physical activity. This phenomenon may be referred to as sensitivity to physical activity (SPA). Indices of SPA may be calculated from the changes in pain responses (intensity, thresholds) from before to after a standardized physical activity (e.g. walking, lifting). Previous research has shown that higher SPA values are associated with greater pain, elevated disability, lower activity tolerance and decreased work output. These studies also showed that SPA as a risk factor predicted these negative outcomes after controlling for other established risk factors. Therefore, high SPA may serve as a valuable tool to identify a priori which patients are most likely to have poor outcomes after a prescribed exercise or physical activity. The ability to identify these individuals before starting treatment may lead to a risk-stratified approach to prescribing exercise interventions, increasing the likelihood of achieving the intended effects of reduced pain and disability. In this presentation, the concept of SPA will be described, as well as its current assessment methods and its potential clinical implications. The presenter will also describe predictors of high SPA: negative pain cognitions and dysfunctional nervous system pain modulation. These factors constitute potential targets for future research investigating interventions aimed at reducing high SPA.

## Speaker 3 Abstract Title: *Encouraging self-management in primary health care through a tailored approach to physical activity and exercise interventions*

**Speaker 3 Name**: Jordan Miller

**Speaker 3 Affiliation**: School of Rehabilitation Therapy, Queen’s University, Kingston, Ontario, Canada

**Speaker 3 Abstract**: Self-management interventions provide an important opportunity to improve functional outcomes in primary health care. However, research suggests most self-management programs improve knowledge and self-efficacy, but produce only small changes in pain or function. In a recent randomized controlled trial, a chronic pain self-management program that incorporated pain neurophysiology education and exercise improved function of participants in comparison to usual care. The effect size of this change in function was greater than that demonstrated in previous trials on self-management for chronic pain (d=0.49 versus d=0.19 to 0.28 in previous studies). One of the key differences in this self-management approach is the inclusion of tailored, rather than standardized, physical activity and exercise. A key hypothesis of the program is that exercise that is tailored to the participant’s goals and sensitivity to physical activity will be more likely to be adhered to and more likely to result in a positive change in pain and function. In this presentation, evidence for a tailored approach to physical activity and exercise will be provided along with a practical self-management strategy that can be implemented in primary health care settings to support people with pain in tailoring their physical activity and exercise based on their own sensitivity to physical activity. This presentation will build on the previous presentation by providing a strategy to tailor physical activity and exercise for people high sensitivity to physical activity that may pose a barrier to participation in standardized physical activity and exercise interventions.

## Learning Objectives

1. Understand that movement, therapeutic exercise, and physical activity should be incorporated in effective pain management.

2. Be able to identify people who have high sensitivity to physical activity which may form a barrier to participating in standardized movement or exercise interventions.

3. Be able to implement a simple approach to help patients tailor their own movement and exercise giving consideration to their response to physical activity.

## Leveraging user-centred design principles to develop and evaluate digital health technologies for pain assessment and self-management

Chitra Lalloo^a,*^, Lynn Cooper^b,*^, Kathryn Birnie^a,c^, and Michael McGillion^d^

^a^The Hospital for Sick Children, Child Health Evaluative Sciences, Toronto, Ontario, Canada; ^b^Canadian Pain Coalition; ^c^University of Toronto, Lawrence S. Bloomberg Faculty of Nursing, Toronto, Ontario, Canada; ^d^McMaster University, Faculty of Health Sciences, Hamilton, Ontario, Canada

**CONTACT** Chitra Lalloo chitra.lalloo@sickkids.ca


^*^Symposium Chairs


© 2017 Chitra Lalloo, Lynn Cooper, Kathryn Birnie, and Michael McGillion. Published with license by Taylor & Francis.

This is an Open Access article distributed under the terms of the Creative Commons Attribution License (http://creativecommons.org/licenses/by/3.0/), which permits unrestricted use, distribution, and reproduction in any medium, provided the original work is properly cited. The moral rights of the named author(s) have been asserted.

Digital health technologies such as smartphone apps, websites, and medical devices, offer new ways to deliver ongoing assessment and self-management support to individuals with painful conditions. These technologies can be leveraged to provide engaging self-management care that is tailored to the needs of different patient groups and individual patients. User-centred design refers to a systematic, iterative process involving assessment of user needs, prototyping, usability evaluation, and deployment. In the context of self-management interventions, “users” often include both patients and healthcare providers. This approach is consistent with patient-oriented research that strives to engage patients as partners, focus on patient-identified priorities, and improve patient outcomes (CIHR, 2016). This symposium will describe approaches to user-centred design that have been successfully applied in patient-oriented research programs. Lessons learned from diverse, funded projects spanning pediatric, young adult, and adult patient populations, each in differing stages of development, will be distilled and discussed. Practical case studies will address a variety of digital delivery platforms, including smartphones, websites, and medical devices. Techniques used to seek and incorporate patient perspectives throughout the intervention design process will be made explicit, along with associated challenges and successful mitigation strategies. The presentations will include embedded user perspectives from both patient partners and healthcare providers (HCPs), integral to each development team. Lynn Cooper (President, Canadian Pain Coalition), involved in all three case studies as a consumer consultant, will act as discussant, offering her perspective on partnerships through the design and evaluation phases of these digital health technologies.

## Speaker 1 Abstract Title: *My post-operative pain (MyPOP): A smartphone-based app to address gaps in post-operative pain self-management for youth*

**Speaker 1 Name**: Kathryn Birnie

**Speaker 1 Affiliation**: The Hospital for Sick Children, Child Health Evaluative Sciences; University of Toronto, Lawrence S. Bloomberg Faculty of Nursing, Toronto, Ontario, Canada

**Speaker 1 Abstract**: Despite evidence-based management and clinical practice standards, moderate to severe post-operative pain is common for the >80,000 youth who undergo surgery in Canada each year. Minimal pain management support is available once youth are discharged from hospital. This is critical to address as poorly managed post-operative pain contributes to negative outcomes, including poor quality of life, increased opioid use, and risk for chronic pain. To address this gap, we are developing “My Post-Operative Pain” or MyPOP, a smartphone-based app to improve self-management of acute post-operative pain in youth aged 8-18 years. The MyPOP app is designed to provide in-the-moment, individually tailored evidence-based pain management advice directly to youth. Methods: Completed steps in the user-centered design process include: (a) needs assessment with patients (n=14), parents (n=13), and healthcare providers (n=15); (b) low fidelity app prototype; and (c) two-day consensus conference with multidisciplinary pediatric post-operative pain experts (n=13). Results: User identified app functions refined during the consensus meeting included pain tracking, goal setting, and pain management algorithm. Two consumers participated as equal members in the consensus meeting, including an adolescent patient, and a consumer organization (Lynn Cooper, President of the Canadian Pain Coalition). Their voices will be represented in this presentation to share their perspectives collaborating in the MyPOP consensus meeting. Future Directions: Details will be shared about remaining phases in the user-centered development of MyPOP, including usability and feasibility testing with adolescent day surgery patients and healthcare providers, and a pilot randomized controlled trial to evaluate implementation and preliminary effectiveness.

## Speaker 2 Abstract Title: *iCanCope: A web and smartphone-based pain self-management platform for youth aged 12-25 years with persistent pain*

**Speaker 2 Name**: Chitra Lalloo

**Speaker 2 Affiliation**: The Hospital for Sick Children, Child Health Evaluative Sciences, Toronto, Ontario, Canada

**Speaker 2 Abstract**: Aim: Persistent pain in adolescents and young adults (AYA) can negatively impact all aspects of health related quality of life. AYA living with persistent pain are expected to assume increasing responsibility for managing their pain. However, the vast majority never receive comprehensive education or coping skills training to promote pain self-management and improve function. This project aims to develop and evaluate iCanCope™, a web- and smartphone-based pain self-management platform tailored for AYA with persistent pain. Condition-specific configurations are being developed for chronic pain, arthritis, and sickle cell disease. Methods: In Phase 1, qualitative needs assessment studies were completed with patients and healthcare providers. A two-day consensus meeting was also held with patients, clinicians, and software developers. In Phase 2, the iCanCope™ prototype was created in collaboration with the Centre for Global eHealth Innovation and AboutKidsHealth. This process involved consultation with knowledge users and healthcare providers. The prototype was refined through iterative usability testing involving semi-structured individual interviews with AYA. Results: The iCanCope™ app includes: (I) symptom self-monitoring in the form of daily check-ins; (II) personalized goal setting; (III) pain coping strategies; and (IV) peer-based social support. The website includes complementary pain self-management education. Usability testing was completed with N=15 AYA (80% female). The prototype was found to be easy to use and understand. Iterative testing identified ways of enhancing the program. Conclusions: iCanCope™ has been developed and deployed by applying the principles of user-centred design. In Phase 3, feasibility will be evaluated through condition-specific pilot randomized controlled trials.

## Speaker 3 Abstract Title: *Getting THE SMArTVIEW CoVerRed (TecHnology Enabled remote automated monitoring and pain Self-MAnagemenT: VIsion for patient EmpoWerment following Cardiac and VasculaR surgery) in Canada and the United Kingdom*

**Speaker 3 Name**: Michael McGillion

**Speaker 3 Affiliation**: McMaster University, Faculty of Health Sciences, Hamilton, Ontario, Canada

**Speaker 3 Abstract**: Aim: Tens of thousands of cardiac and vascular surgeries (CaVS) are performed on seniors in Canada and the United Kingdom (UK) each year to improve survival, relieve disease symptoms, and improve health-related quality of life (HRQL). However, multiple challenges threaten patient safety including: transition to chronic post-surgical pain (CPSP), surgical site infection, undetected hemodynamic compromise, and related poor functioning. The purpose of this study is to test usability of an eHealth-enabled service delivery intervention, SMArTVIEW, which combines remote automated monitoring and virtual self-management recovery support following CaVS. As a connected care strategy, SMArTVIEW is framed by the ‘Patients First’ Action Framework and was co-designed by seniors who suffered postoperative adverse events. Methods: ‘Out-of-the-box’ user testing, underway, is designed for Health Canada approved devices built for scale. Eighteen CaVS nurses and patients are being observed while performing required SMArTVIEW user tasks during high-fidelity simulation. Observations are being documented according to an adapted, patient co-designed user testing rubric. Participants are invited to think aloud in order to distill system navigation issues/problems, areas of frustration, or confusion around workflow. User testing commenced Nov 25 2016 and ends March 15 2017. Results: Results reported will include user task time to completion; proportion of use-errors, distraction points, difficulties encountered, root causes of problems; and use-related behaviours. Significance: This unique approach to user testing represents a formalized method for engagement of patients and HCPs in optimization of workflow in order to inform the detailed protocol of a major SMArTVIEW clinical trial in Canada and UK (2017-2020).

## Learning Objectives

1. Explain three different user-centred design approaches, and appraise the advantages and disadvantages of each approach.

2. Illustrate specific examples of how patient and HCP engagement significantly impacted the iterative design process and usability of digital self-management interventions.

3. Integrate “lessons learned” from pediatric and adult pain use case scenarios to inform their own digital health initiatives, going forward.

## Unravelling the Relationship between Sleep and Pain: A Translational, Lifespan Perspective

Patrick Finan^a,*^, Melanie Noel^b^, and Richelle Mychasiuk^c^

^a^Johns Hopkins University, School of Medicine, Baltimore, MD, USA; ^b^University of Calgary and Alberta Children’s Hospital Research Institute (Behavior and the Developing Brain Theme), Calgary, Alberta, Canada; ^c^University of Calgary and Hotchkiss Brain Institute, Calgary, Alberta, Canada

**CONTACT** Patrick Finan pfinan1@jhu.edu


^*^Symposium Chair


© 2017 Patrick Finan, Melanie Noel, and Richelle Mychasiuk. Published with license by Taylor & Francis.

This is an Open Access article distributed under the terms of the Creative Commons Attribution License (http://creativecommons.org/licenses/by/3.0/), which permits unrestricted use, distribution, and reproduction in any medium, provided the original work is properly cited. The moral rights of the named author(s) have been asserted.

The relationship between sleep and pain is well established and robust. Epidemiological studies have repeatedly shown that sleep impairment predicts both the development and worsening of pain problems in children and adults. While previous theoretical and empirical work supported a bidirectional relationship between sleep and pain, more recent evidence suggests that sleep is a stronger predictor of pain. At a neurobiological level, sleep impairment has been shown to disrupt pain inhibitory processes, leading to the development and maintenance of pain problems. At a behavioral level, impairments in subjective and objective sleep quality are associated with deficits in mood and cognition, which confer risk for worsening pain trajectories. Indeed, the coupling between depression and pain may be partially explained by variation in sleep continuity and architecture. These relationships have important clinical implications. Sleep disturbances have been shown to impair response to psychosocial pain treatments, making it an increasingly important (yet underutilized) target in pain management interventions. Nevertheless, important questions remain regarding mechanisms underlying the sleep-pain relationship and its impact across development.

This workshop will present new evidence demonstrating the role of sleep in the experience of acute and chronic pain in pediatric and adult populations and across human and rodent studies. The panel is comprised of a group of clinical and basic scientists who utilize a range of innovative methodological approaches to assess sleep (e.g., sleep deprivation, assessment of circadian rhythms using telemetry probes, polysomnography, actigraphy, daily sleep diary) and pain (e.g., quantitative sensory testing, self-report, functional magnetic resonance imaging).

## Speaker 1 Abstract Title: *The effects of sleep disruption on measures of pain inhibition and facilitation in adults*

**Speaker 1 Name**: Patrick Finan

**Speaker 1 Affiliation**: Johns Hopkins University School of Medicine, Baltimore, MD, USA

**Speaker 1 Abstract**: Patients with chronic pain rarely report static levels of pain from day to day. Rather, pain tends to ebb and flow on a daily basis. A large body of evidence suggests that affective (e.g., positive and negative affect), cognitive (e.g., pain catastrophizing), and neurobiological (e.g. descending opioidergic and dopaminergic pain modulation) factors influence the daily experience of pain through both inhibitory and facilitative mechanisms. Recent studies demonstrate that sleep disruption (i.e., nocturnal awakenings after sleep onset) alters affect, cognition, and neural circuitry in a manner that promotes increased pain sensitivity. Dr. Finan will interrogate the extent to which sleep disruption alters pain inhibitory and facilitative processes. To that end, he will present recent and unpublished data from both experimental sleep deprivation studies in healthy participants and clinical studies of patients with chronic pain. Specifically, he will present data from a within-person crossover experiment comparing the effects of repeated nocturnal awakenings to normal sleep on functional magnetic resonance imaging measures of pain inhibition and facilitation in healthy young adults. In synergy with Dr. Noel’s presentation on pediatric patients, Dr. Finan will present data from middle-aged and older adults with temporomandibular joint disorder and knee osteoarthritis, respectively, that will explicate the effects of daily spikes in positive and negative affect on clinical pain severity, and the extent to which nocturnal sleep disruption alters those day-to-day measures of pain inhibition and facilitation. Together, these presentations will offer a picture of how sleep modulates cognitive and affective mediators of pain throughout the lifespan.

## Speaker 2 Abstract Title: *The relationship between sleep and acute and chronic pediatric pain*

**Speaker 2 Name**: Melanie Noel

**Speaker 2 Affiliation**: University of Calgary and Alberta Children’s Hospital Research Institute (Behavior and the Developing Brain Theme), Calgary, Alberta, Canada

**Speaker 2 Abstract**: Emerging evidence suggests that sleep is integral to the experience of pain in childhood and adolescence. In the context of pediatric surgery, sleep is often disrupted after discharge and negatively influences post-surgical pain trajectories. In the context of pediatric chronic pain, insomnia symptoms are reported with high frequency in adolescents and impede outcomes and response to pain management interventions. In a recently proposed conceptual model of co-morbid mental health issues and pediatric chronic pain, sleep was proposed as a key underlying mechanism. Moreover, sleep disruption is thought to lead to distortions in pain-related cognitions (e.g. pain memory), which are powerful predictors of subsequent pain experience. Dr. Noel will present new data demonstrating the role of sleep in the development of pain memory biases and pain trajectories in cohorts of young children (aged 4-7 years) undergoing surgery. She will present preliminary data investigating positively valenced affective and social mediators of the effect of sleep disturbance on negative pain memory. In separate samples of treatment seeking adolescents with chronic pain, the mechanistic role of poor subjective sleep quality in the relationship between mental health comorbidities and pain complaints and impairment will also be demonstrated. Finally, Dr. Noel will present data on the role of sleep impairment on functional pain outcomes in youth with chronic pain from intake to several months later. Implications for treatment development and tailoring will be discussed.

## Speaker 3 Abstract Title: *The effects of pain on disruption of circadian rhythms following mTBI in adolescent rats*

**Speaker 3 Name**: Richelle Mychasiuk

**Speaker 3 Affiliation**: University of Calgary and Hotchkiss Brain Institute, Calgary, Alberta, Canada

**Speaker 3 Abstract**: Children and adolescents have the highest rates of traumatic brain injury (TBI) resulting in hospital visits, with mild and repetitive mild TBI (RmTBI) accounting for 80% of these injuries. Head pain is one of the primary symptom complaints in this population. What’s more, adolescents are disproportionately represented in populations at risk for sleep disturbances and sleep deprivation. Current guidelines recommend between 9-11 hours of sleep for adolescents, but they average < 7, and more than 1/3 of teenagers report falling asleep at school. Given the fundamental relationships between neuroplasticity, sleep, and pain, we sought to determine if pain tolerance in rodents was associated with disruptions in circadian rhythms following mTBI. Dr. Mychasiuk will present new data demonstrating that mTBI in adolescence disrupts daily circadian rhythms and reduces the amount of time rats spend asleep. In addition, she will present data that associates the rat’s pain sensitivity before and after mTBI with circadian rhythm disruption.

## Learning Objectives

1. To evaluate the role of sleep as an upstream predictor of pain inhibitory and facilitative processes throughout the lifespan.

2. To examine the role of sleep in the development of cognitive biases and post-surgical pain in young children as well as its role in trajectories of pain and function in adolescents with chronic pain.

3. To examine the relationship between circadian rhythms and pain responsivity in adolescent rats following mild traumatic brain injury.

## Helping the Complex Chronic Pain Patient: A Multidisciplinary Approach to Reversing CNS Hyperexcitability

Joel Katz 0000-0002-8686-447X ^a,b,*^, Aliza Weinrib^a,b^, Linda Cundiff^c^, Jessica Dulong^c^, and Hance Clarke^b^

^a^Department of Psychology, York University, Toronto, ON, Canada; ^b^Transitional Pain Service, Department of Anesthesia and Pain Management, Toronto General Hospital, Toronto, ON, Canada; ^c^Pain Program, Royal Jubilee Hospital, Victoria, BC, Canada

**CONTACT** Joel Katz jkatz@yorku.ca


^*^Symposium Chair


© 2017 Joel Katz, Aliza Weinrib, Linda Cundiff, Jessica Dulong, and Hance Clarke. Published with license by Taylor & Francis.

This is an Open Access article distributed under the terms of the Creative Commons Attribution License (http://creativecommons.org/licenses/by/3.0/), which permits unrestricted use, distribution, and reproduction in any medium, provided the original work is properly cited. The moral rights of the named author(s) have been asserted.

This symposium will feature speakers from four disciplines whose clinical practices are all aimed at eliminating the ravages of chronic pain and in particular at reducing the psychological, physical, and physiological correlates of central neural mechanisms that have gone awry and that often accompany and contribute to the hypersensitivity evident in many chronic pain conditions. The session begins with a presentation from Aliza Weinrib, lead psychologist at the Toronto General Hospital, Transitional Pain Service who will illustrate with examples two approaches she uses to reducing sympathetic nervous system over-reactivity including relaxation training and Acceptance and Commitment Therapy (ACT). This is followed by a joint presentation by occupational and physical therapists, Linda Cundiff and Jessica Dulong, from the Pain Program at the Royal Jubilee Hospital in Victoria who will illustrate their approach to “calming the nervous system” in a lively session involving helpful tips, pointers and activities. They will bring to light how everyday encounters with clients can be used in the service of reducing CNS hypersensitivity and how physical activity can be incorporated into the treatment plan to foster self-confidence and an active re-engagement in life. The session will conclude with a presentation from Hance Clarke, anesthesiologist and Director of Pain Services at the Toronto General Hospital who will wrap-up the session and briefly discuss pharmacological management designed to reduce CNS hyperexcitability.

## Speaker 1 Abstract Title: *Psychological strategies to reduce CNS hyperexcitability*

**Speaker 1 Name**: Aliza Weinrib

**Speaker 1 Affiliation**: Transitional Pain Service, Department of Anesthesia and Pain Management, Toronto General Hospital, Toronto, Ontario, Canada

**Speaker 1 Abstract**: This presentation will outline and contrast two psychological approaches to calming the sympathetic nervous system and activating the parasympathetic nervous system for patients living with complex, persistent pain. The first approach involves inducing the relaxation response, which has many benefits for physical and psychological health, and has been a mainstay of psychological intervention for pain for many years. Training patients to induce the relaxation response has the goal of reducing nervous system activation, and consequently reducing central amplification of pain. The second approach is mindfulness and acceptance training for patients living with pain, and can utilize (a) mindfulness meditation techniques and (b) behavioral strategies based on Acceptance and Commitment Therapy (ACT). The goal of mindfulness and acceptance-based approaches is to “allow,” “open,” or “make space” for pain sensations, leading to reduced psychological reactivity to pain over time. This presentation will review key relaxation and mindfulness interventions, as well as metaphors and experiential exercises used in ACT. We will share the patient perspective on these techniques via a patient video, as well as share some clinical data from our application of mindfulness and ACT with complex pain patients in the post-surgical Transitional Pain Service at Toronto General Hospital.

## Speaker 2 Abstract Title: *Myths and movement*

**Speaker 2 Name**: Linda Cundiff and Jessica Dulong

**Speaker 2 Affiliation**: Pain Program, Royal Jubilee Hospital, Victoria, British Columbia, Canada

**Speaker 2 Abstract**: We begin by addressing how professionals amplify pain cycles and chronicity in our clients. Medical language, speed of interactions and top-down hierarchal approaches maintain and reinforce our clients’ beliefs in the acute model of pain in which they are led to believe that chronic pain arises from tissue damage and that they must be cured before they can get on with their lives. We will describe and illustrate how we educate our clients regarding building calm nervous systems, and how their 70% contribution to their pain recovery gives them an active engaged perspective when living with pain. Professionals modelling a calm nervous system encourage clients to move in easeful and confident patterns that build new “neurotags” which serve to de-amplifiy their sensitized neurobiological systems and ensures we are truly working in a broad biopsychosocial model.

## Speaker 3 Abstract Title: *The physician’s role in treating complex pain patients and reducing CNS hyperexcitability*

**Speaker 3 Name**: Hance Clarke

**Speaker 3 Affiliation**: Director, Transitional Pain Service, Department of Anesthesia and Pain Management, Toronto General Hospital, Toronto, Ontario, Canada

**Speaker 3 Abstract**: Inadequate pain management can have significant impacts on a patients’ quality of life. The prescription pad can be a helpful tool and this session will highlight central nervous system (CNS) drugs aimed at reducing CNS hyperexcitability and treating complex pain patients. Data will be shared which demonstrates medications helpful with respect to opioid weaning strategies and pain management. Although the prescription pad can be helpful, the physicians’ role in the management of a complex pain patient is often overstated. Physicians must take care with respect to their clinical approach with these individuals, building on the previous two presentations, this session will extrapolate on the facilitative role that physicians should assume while supporting multidisciplinary pain teams.

## Learning Objectives

1. Participants will be able to classify and describe psychological techniques that can calm the nervous system in patients living with complex pain.

2. Participants will understand the impact health professionals have on clients’ nervous system and how clients can realize the huge potential in developing a calming and healing nervous system.

3. To discuss medications that impact the CNS in pain patients and understand the physician’s role in reducing CNS hyperexcitability.

## Pain as a sensory disturbance: manipulation, modulation and mechanisms

Massieh Moayedi 0000-0002-7324-2540 ^a,*^, Jenny Lewis^b^, and Janet Bulititude^c^

^a^Faculty of Dentistry, University of Toronto, Toronto, Ontario, Canada; ^b^Royal National Hospital for Rheumatic Diseases, Upper Borough Walls, Bath, United Kingdom; ^c^Psychology Department University of Bath, Bath, United Kingdom

**CONTACT** Massieh Moayedi m.moayedi@dentistry.utoronto.ca


^*^Symposium Chair


© 2017 Massieh Moayedi, Jenny Lewis, and Janet Bulititude. Published with license by Taylor & Francis

This is an Open Access article distributed under the terms of the Creative Commons Attribution License (http://creativecommons.org/licenses/by/3.0/), which permits unrestricted use, distribution, and reproduction in any medium, provided the original work is properly cited. The moral rights of the named author(s) have been asserted.

Sensory perceptual abnormalities associated with pain can occur in chronic pain conditions and Complex Regional Pain Syndrome (CRPS) is an exemplary model.

This workshop gives an overview of the evidence for sensory perceptual disturbances in chronic pain and how these can be modulated for pain relief. We will present findings from recent CRPS research that explores the use of visual illusions in normalising the appearance of the painful hand, followed by studies that measure and manipulate spatial attention. These will be discussed in the context of how sensory and perceptual abnormalities can be manipulated for therapeutic benefit.

Finally, we will present novel neuroimaging data to provide insight in the underlying neural mechanisms associated with these perceptual changes

## Speaker 1 Abstract Title: *Visual manipulation, pain modulation: Exploring the use of hand illusions in CRPS*

**Speaker 1 Name**: Dr. Jenny Lewis

**Speaker 1 Affiliation**: Royal National Hospital for Rheumatic Diseases, Upper Borough Walls, Bath, United Kingdom

**Speaker 1 Abstract**: People with Complex Regional Pain Syndrome (CRPS) dislike the appearance of their painful limb, a characteristic feature of body perception disturbance. Growing evidence suggests that an illusionary change to the painful body part appears to reduce pain. We hypothesized that repeated exposure to visual illusions created by the MIRAGE system, a form of augmented virtual reality to change hand appearance, would reduce hand pain in CRPS. We performed a study of 38 people with CRPS of one arm were randomly allocated in a blinded manner to either a control or experimental group. While both hands were placed in the MIRAGE system, the experimental intervention involved digitally changing the visual appearance of the affected hand according to the patient’s description of how they wished their hand to look. Participants were exposed to the resultant image for one minute per session for a total of four sessions. Numeric rating of current hand pain was reported pre- and post-intervention. We found a significant reduction in hand pain following repeated illusionary exposure when compared to controls. These data suggest that repeated exposure to illusions that visually altered the appearance of the hand provided greater pain reductions than a single exposure. These findings support the need for further research to determine the optimum dose for therapeutic use.

## Speaker 2 Abstract Title: *Spatial cognition in Complex Regional Pain Syndrome*

**Speaker 2 Name**: Dr. Janet Bulititude

**Speaker 2 Affiliation**: Psychology Department University of Bath, Bath, United Kingdom

**Speaker 2 Abstract**: Two hypotheses about the neurological bases of CRPS are that symptoms arise due to mismatch between sensory input, motor output and movement intention; and that patients have a deficit in attending to—or “neglect” of—the affected side of space. I will present evidence for each of these hypotheses from two studies.

First, seven upper- and seven lower-limb CRPS patients completed a task in which they indicated the position of a target under three sensory conditions: visual-only (V) guidance, motor-only (M) guidance, and combined visual and motor (V-M) guidance (the “sensory-motor integration” condition). The spatial scatter of end points (pooled across X and Y directions) indicated that unlike controls, patients were not significantly more accurate in the V-M condition compared to the V and M conditions. That is, the normal advantage that is experienced under conditions of multisensory guidance was not achieved by CRPS patients, indicating a deficit in sensory-motor integration.

Second, we used a sensitive test called the temporal order judgement task to examine the distribution of visual attention in twelve upper- and twelve lower-limb CRPS patients. Two lights were projected to the left and right of a fixation point at different temporal offsets. The participants indicated which appeared first. CRPS patients were slower to process visual stimuli on the affected relative to the unaffected side of space, indicating inattention to the affected side.

I will discuss these results with reference to the potential utility a sensory-motor illusion, called “prism adaptation” in the treatment of CRPS.

## Speaker 3 Abstract Title: *Neural correlates of visuo-haptic illusions: Potential mechanisms of pain modulation*

**Speaker 3 Name**: Dr. Massieh Moayedi

**Speaker 3 Affiliation**: Faculty of Dentistry, University of Toronto, Toronto, Ontario, Canada

**Speaker 3 Abstract**: The perception of our body can easily be manipulated, as evidenced by a variety of illusions that change the shape of the body, demonstrating that the manner in which the body is perceived is highly adaptive. These illusions disrupt the body schema—a virtual, dynamic, multisensory map of the body within the sensory environment. Visual illusions, such as the rubber hand illusion and the mirror box, are commonly used to treat chronic pain disorders.These illusory disruptions can be reinforced by simultaneously manipulating two or more sensory channels. For example, reinforcing a visual illusion with haptic stimulation substantially enhances the robustness of the illusion, and the rates of susceptibility. The combination of visual and tactile information requires multisensory processing and binding in higher order cortical regions. However, the neural correlates of a visuohaptic illusion performed on subject’s own limb have yet to be investigated. Here, we present novel data about the brain networks related to a visuohaptic illusions, and the potential mechanisms by which they can affect pain perception.

## Learning Objectives

1. Complex regional pain syndrome is a disease of the CNS, affecting cognitive, spatial and sensorimotor regions.

2. Noninvasive approaches, such as visual illusions, can affect the ownership of and attention to the affected limb in CRPS, and reduce clinical, spontaneous pain

3. Understand that visual illusions affect the image of the limb in the CNS, and activate top-down controls that modulate pain

## Is it time to replace sweet solutions with sweet parents? Parental led interventions as the standard of care for procedural pain management in infants

Marsha Campbell-Yeo^a,*^, Manon Ranger^b^, and Jack Hourigan^c^

^a^School of Nursing, Departments of Pediatrics, Psychology and Neuroscience, Dalhousie University and IWK Health Centre, Halifax, Nova Scotia, Canada; ^b^Nurture Science Program, Department of Psychiatry, Division of Developmental Neuroscience Columbia University, New York, USA; University, New York, Canada; ^c^Toronto, Ontario, Canada

**CONTACT** Marsha Campbell-Yeo marsha.campbellyeo@iwk.nshealth.ca


^*^Symposium Chair


© 2017 Marsha Campbell-Yeo, Manon Ranger, and Jack Hourigan. Published with license by Taylor & Francis.

This is an Open Access article distributed under the terms of the Creative Commons Attribution License (http://creativecommons.org/licenses/by/3.0/), which permits unrestricted use, distribution, and reproduction in any medium, provided the original work is properly cited. The moral rights of the named author(s) have been asserted.

Sweet tasting solutions, such as sucrose or glucose, are currently considered the gold standard for the treatment of needle related and routinely performed minor procedural pain in both full term and prematurely born neonates. However, despite strong evidence of the effectiveness to reduce behavioural pain response and recommendations that its non use would be unethical, some concerns have been raised regarding the possibility of adverse effects following repeated exposure on the developing brain of the youngest and sickest neonates. Moreover, the efficacy of sucrose to reduce cortical responses associated with procedural pain exposure in newborns has yet to be demonstrated. As such, alternative treatments are continuing to be explored. Thus, involvement of parents in neonatal pain management is of increased interest in both research and clinical settings. From an evolutionary view, the mother is the optimal source of physical and psychological support for the infant, both as a fetus and after birth. Hospital care and medical interventions are sources of separation and stress, leading to a diminished capacity for the infant to endure painful procedures and situations.

In this workshop, utilizing basic and clinical science as well as parent perspectives, we suggest redefining the hierarchy of pain treatment from sweet tasting solutions to parents as the consistent first line treatment for infant pain relief. This implies a change of role of parents, from being present or being advocates for their infant, to being active partners in pain care. We will discuss the evidence behind this recommendation, and listen to the viewpoint of a parent of a preterm infant regarding this new role, including the challenges and opportunities to engage families.

Intended audience: Clinicians and researchers with interest in neonatal pain management and knowledge translation.

## Speaker 1 Abstract Title: *Let’s not sugar coat it: Evidence from a translational pain mouse model of long term effects of repeated sucrose for pain management in very preterm infants*

**Speaker 1 Name**: Manon Ranger

**Speaker 1 Affiliation**: Associate Research Scientist, Nurture Science Program Department of Psychiatry, Division of Developmental Neuroscience Columbia University, New York, USA

**Speaker 1 Abstract**: Effective pain management is crucial to help mitigate the short/long-term negative consequences of early pain exposure in very preterm infants. However, the optimal strategy to achieve this goal is not clear. Currently, oral sucrose is administered routinely to reduce pain of minor procedures and is recommended as standard care in international guidelines, which are based on evidence of safety and efficacy of a single dose of sucrose to reduce pain behaviours. Long-term benefits or risks of repeated sucrose administration on brain development and neurodevelopmental outcomes in the preterm population have not been evaluated. Since oral sucrose is now used extensively, clinical equipoise no longer exists for randomized controlled trials of sucrose use for pain. Recently our group used a translational animal model to explore these questions. In this context, we examined the effects of neonatal repetitive sucrose exposure on brain development in a mouse pain model that closely mimics NICU care. Mice make an excellent preclinical model to study conditions in the human preterm neonate because they are born neurologically immature, with the first postnatal week in the mouse corresponding to the development of the human limbic system, striatum and cerebellar development at 24-32 weeks gestation. In this symposium, current evidence from our translational studies will be presented through recent findings in neuroimaging and behavioural function. Given that our results raise concerns for the use of sucrose as a standard pain management practice in the very preterm population, we anticipate a lively discussion between clinicians, researchers and neuroscientists to follow.

Nothing to disclose.

## Speaker 2 Abstract Title: *Mamma really does know best: Evidence update of maternal-led interventions to reduce behavioural pain response in preterm infants*

**Speaker 2 Name**: Marsha Campbell-Yeo

**Speaker 2 Affiliation**: Neonatal Nurse Practitioner, Associate Professor and Clinician Scientist, School of Nursing, Departments of Pediatrics, Psychology and Neuroscience, Dalhousie University and IWK Health Centre, Halifax, Nova Scotia, Canada

**Speaker 2 Abstract**: Despite high ubiquitous pain exposure in hospitalized neonates, an average of 12 painful procedures per day, less than half of neonates receive any pain relieving intervention. In addition to immediate suffering, this poorly treated exposure to early pain is associated with long lasting deleterious outcomes affecting cognitive, physical, and emotional development. Quality evidence suggests that healthcare provider led interventions (e.g. sweet tasting solutions) and parent-led (e.g. breastfeeding and skin-to-skin contact [SSC]), and are most effective; however, clinicians and parents often struggle with which strategy should be recommended as first line therapy or whether several should be given in combination. Findings from our recently updated 2016 Cochrane review, including the results of a GRADE assessment, related to the efficacy of SSC to reduce procedural pain in newborns will be presented. In addition to this evidence justifying the use of SSC during a single procedure, data from our recent clinical trial (TRAKC NCT01561547) of 242 preterm infants randomized to SSC + placebo or SSC + sucrose or sucrose alone will be reported, related to sustained efficacy as measured by composite pain scores over repeated procedures throughout an infant’s entire hospitalization. Secondary outcomes include association with neurodevelopment scores at 32 and 26 weeks corrected gestational age. Presentation of these findings will support advocating for parent-led interventions as first line therapy in the management of newborn pain over repeated procedures.

Funded by the Canadian Institutes of Health Research (CIHR) #244640

Nothing to disclose.

## Speaker 3 Abstract Title: *Parenting isn’t a pain in the NICU: Supporting active parental participation in neonatal pain management*

**Speaker 3 Name**: Jack Hourigan

**Speaker 3 Affiliation**: Toronto, Ontario, Canada

**Speaker 3 Abstract**: Consistent, active parental participation in the pain management care of medically fragile neonates is critical to the long-term health and wellbeing of NICU infants and their families. Recognizing how parents perceive pain management, understanding ways to engage parents in care in a practical way, and developing creative communication strategies to disseminate evidence based pain management education to families will increase investment and ensure parental integration into the child’s circle of care.

As the first parent to participate in the Family Integrated Care (FICare) NICU pilot study at Toronto’s Mount Sinai Hospital with her premature (27 weeks gestation) daughter Tess, Ms. Jack Hourigan has lived neonatal pain management experience. Having participated in over 370 hours of skin-to-skin contact, extended breastfeeding and daily NICU evidence based education sessions, she understands the vital role parents play in pain management therapy for their neonates during hospitalization and post discharge. In her former role as Mount Sinai’s NICU Parent Advisor and National Parental co-ordinator in the FICare expansion project, and currently as a healthcare communications specialist and peer support facilitator with the Canadian Premature Babies Foundation, she develops tangible parent engagement strategies and provides clinicians with much needed insight into the parental perspective in the NICU population. In this workshop, participants will learn tangible ways to gain parental trust, support advocacy and reach families through creative education for optimal understanding of the benefits of pain management.

Disclosure:

As a healthcare consultant Jack Hourigan and Sway Partners has gained payment and provided communications services (via workshops, coaching and speaking) to various hospital sites, industry and individuals, but has no sponsorship or interests in this workshop.

## Learning Objectives

1. Describe most recent evidence on long-term effects of early repetitive sucrose exposure on brain and behavioural outcomes in a mouse pain model.

2. Describe the current evidence for parent-led pain relieving interventions available for infants.

3. Recognize and better understand parent perceptions regarding the role of parents in improving pain treatment in hospitalized infants and identify strategies for engaging parents as active participants in infant pain care.

## Cannabis update: from pain to policy

Mark A. Ware^a,*^, Jason Busse^b^, and Zach Walsh^c^

^a^Departments of Anesthesia and Family Medicine, McGill University, Montreal, Quebec, Canada; ^b^Department of Anesthesia, McMaster University, Hamilton, Ontario, Canada; ^c^Department of Psychology, The University of British Columbia, Vancouver, British Columbia, Canada

**CONTACT** Mark A. Ware mark.ware@mcgill.ca


*Symposium Chair


© 2017 Mark A. Ware, Jason Busse, and Zach Walsh. Published with license by Taylor & Francis.

This is an Open Access article distributed under the terms of the Creative Commons Attribution License (http://creativecommons.org/licenses/by/3.0/), which permits unrestricted use, distribution, and reproduction in any medium, provided the original work is properly cited. The moral rights of the named author(s) have been asserted.

The medical use of cannabis continues to be a hot topic in medical and non-medical circles, with increasing interest in policies of non-medical use in Canada. There are increasing concerns about opioid-related harms, and interest in the potential opioid sparing effects of cannabis and cannabinoids. This workshop will review the current level of evidence for the use of cannabinoids in pain, will review the mental health effects of cannabis, and will consider the interactions between opioids and cannabioids in preclinical and clinical studies and practice. Attendees will be invited to share their perspectives and experiences in a dynamic and evidence based workshop.

## Speaker 1 Abstract Title: *Effectiveness of medical marijuana of chronic non-cancer pain: A systematic review*

**Speaker 1 Name**: Jason Busse

**Speaker 1 Affiliation**: Associate professor, Department of Anesthesia, McMaster University, Hamilton, Ontario, Canada

**Speaker 1 Abstract**: This presentation will present a synthesis of all randomized controlled trials that have explored the efficacy and safety of medical marijuana for chronic non-cancer pain. We will explore for sub-group effects across modes of administration (e.?g. smoked, tablet). We will use the GRADE approach to establish the certainty of evidence for each outcome, and use novel methods to present treatment effects in order to optimize interpretability. Our review will provide critical guidance regarding the role of medical cannabis in the management of chronic non-cancer pain.

## Speaker 2 Abstract Title: *Medical cannabis and mental health: A guided systematic review*

**Speaker 2 Name**: Zach Walsh

**Speaker 2 Affiliation**: Associate Professor, Department of Psychology, The University of British Columbia, Vancouver, British Columbia, Canada

**Speaker 2 Abstract**: This talk considers the potential influences of the use of cannabis for therapeutic purposes (CTP) on areas of interest to mental health professionals, with foci on psychological intervention and assessment. We identified 31 articles relating to CTP use and mental health, and 29 review articles on cannabis use and mental health that did not focus on use for therapeutic purposes. Results reflect the prominence of mental health conditions among the reasons for CTP use, and the relative dearth of high-quality evidence related to CTP in this context, thereby highlighting the need for further research into the harms and benefits of medical cannabis relative to other therapeutic options. Preliminary evidence suggests that CTP may have potential for the treatment of PTSD, and as a substitute for problematic use of other substances. Extrapolation from reviews of non-therapeutic cannabis use suggests that the use of CTP may be problematic among individuals with psychotic disorders. The clinical implications of CTP use among individuals with mood disorders are unclear. With regard to assessment, evidence suggests that CTP use does not increase risk of harm to self or others. Acute cannabis intoxication and recent CTP use may result in reversible deficits with the potential to influence cognitive assessment, particularly on tests of short-term memory.

## Speaker 3 Abstract Title: *Putting it all together: The role of pain medicine in a legal cannabis future*

**Speaker 3**: Mark A. Ware

**Speaker 3 Affiliation**: Associate Professor, Departments of Anesthesia and Family Medicine, McGill University, Montreal, Quebec, Canada

**Speaker 3 Abstract**: Access to cannabis for non-medical is about to become a reality in Canada. This workshop session will consider several important implications of this new system on pain medicine: the role of the health care professional, the need for research, and the evolving evidence for the interaction between cannabinoids and opioids.

## Learning Objectives

1. Review the evidence for cannabis and cannabinoid in pain management

2. Review the evidence for the effects of cannabis on mental health

3. Consider the role of the pain profession in a legal cannabis environment

## Pain Scales Development and Validation across Age, Culture and Clinical Contexts of Care

Sylvie Le May^a,*^, Ariane Ballard^a^, Margot Latimer^b^, John R. Sylliboy^b^, and Céline Gélinas^c^

^a^Faculty of Nursing, University of Montreal, CHU Ste-Justine Research Centre, Montreal, Quebec, Canada; ^b^Dalhousie University Faculty of Health Professions, Pediatric Pain Research Centre IWK Health Centre, Halifax, Nova Scotia, Canada; ^c^McGill University, Ingram School of Nursing Center for Nursing Research and Lady Davis Institute Montreal, Quebec, Canada

**CONTACT** Sylvie Le May sylvie.lemay@umontreal.ca


^*^Symposium Chair


© 2017 Sylvie Le May, Ariane Ballard, Margot Latimer, John R. Sylliboy, and Céline Gélinas. Published with license by Taylor & Francis

This is an Open Access article distributed under the terms of the Creative Commons Attribution License (http://creativecommons.org/licenses/by/3.0/), which permits unrestricted use, distribution, and reproduction in any medium, provided the original work is properly cited. The moral rights of the named author(s) have been asserted.

Instruments are essential for clinicians and researchers to assess pain and provide personalized pain management. However, pain is an inherently subjective experience. Therefore, scales selected should have been validated with the targeted population prior to their specific use. This workshop aims to present the process of development or validation of selected pain measurement tools across different age-groups, culture and clinical contexts. Three researchers interested by the development and psychometric testing of pain scales will each present their different projects thus research on self-report pain measures in children using existing scales, newly developed applications for Aboriginal children and combination of physiological measures to better assess pain in critically ill adult patients. Participants will gain more knowledge on psychometric properties of existing scales as well as learn about new applications and technologies to assess pain.

## Speaker 1 Abstract Title: *Comparisons of psychometric properties of the VAS, FPS-R and CAS used in children with acute pain within an emergency department context*

**Speaker 1 Name**: Ariane Ballard

**Speaker 1 Affiliation**: Faculty of Nursing, University of Montreal, CHU Ste-Justine Research Centre, Montreal, Quebec, Canada

**Speaker 1 Abstract**: Introduction. Appropriate pain management relies on the use of valid, reliable and age-appropriate tools that are validated in the setting in which they are intended to be used. The aim of the study was to assess the psychometric properties of three pain scales used for children presenting to the ED with a musculoskeletal injury.

Methods. Convergent validity was assessed by determining the Pearson’s correlations and the agreement using the Bland-Altman method between VAS, FPS-R and CAS. Responsiveness to change was determined by performing the Wilcoxon signed-rank test between the pre-post analgesia mean scores. Reliability of the scales was estimated using relative (Pearson’s correlation, ICC) and absolute indices (CR).

Results. A total of 495 participants was included in the analyses. Mean age of 11.9 ± 2.7 y.o. and mainly boys (55.3%). Correlation between each pair of scales was 0.78 (VAS/FPS-R), 0.92 (VAS/CAS) and 0.79 (CAS/FPS-R). Limits of agreement (80%CI) were -2.71 to 1.27 (VAS/FPS-R), -1.13 to 1.15 (VAS/CAS) and -1.45 to 2.61 (CAS/FPS-R). Responsiveness to change was demonstrated by significant differences in mean pain scores, among the three scales, between pre- and post-medication administration (p<0.0001). ICC and CR estimates suggested acceptable reliability for the three scales at 0.79 and ±1.49 for VAS, 0.82 and ±1.35 for CAS, and 0.76 and ±1.84 for FPS-R.

Conclusion. The scales demonstrated good psychometric properties with a sample of children with acute pain in the ED. The VAS and CAS showed a stronger convergent validity, while FPS-R was not in agreement with the other scales.

## Speaker 2 Abstract Title: *Developing and testing an electronic pain app for aboriginal children & youth*

**Speaker 2 Name**: Margot Latimer and John R. Sylliboy

**Speaker 2 Affiliation**: Dalhousie University Faculty of Health Professions, Pediatric Pain Research Centre IWK Health Centre, Halifax, Nova Scotia, Canada

**Speaker 2 Abstract**: Aboriginal children and youth have some of the highest rates of pain related conditions including ear pain, dental pain and juvenile arthritis. They are also less likely to be treated for these conditions and less likely to have their pain managed appropriately. Our research has identified that Aboriginal children and youth experience and convey their pain differently than non-Aboriginal children due to a range of historical (residential schooling) experiences and cultural traditions.

We have developed an electronic pain app to act as a communication aid in the health care setting for Aboriginal children and youth. The current prototype allows a child to describe their physical pain and tell their pain story using a series of interactive icons. An emotional pain component is being added and the initial testing phase of this addition will be highlighted in this symposia piece as well as future testing plans.

Initial content validity testing was completed with children/youth (n=18) in two Nova Scotia First Nation communities to determine icon style, avatar preference, emotions to include and preferred measurement scales. Three cycles of low fidelity testing will then take place with screen shots of the projected design being shown to children/youth in order to get feedback on design, premise and question format. After each cycle, changes will be implemented and updated screenshots will be shown to the next group. Once a fully developed app is created additional high fidelity testing, content validity, testing of questions and clinical feasibility testing will take place.

## Speaker 3 Abstract Title: *Physiologic Indicators for the assessment of acute pain: Recent evidence and new trends*

**Speaker 3 Name**: Céline Gélinas

**Speaker 3 Affiliation**: McGill University Ingram School of Nursing, Center for Nursing Research and Lady Davis Institute Montreal, Quebec, Canada

**Speaker 3 Abstract**: The assessment of acute pain is challenging especially in patients unable to self-report. Although recommendations and guidelines strongly support the use of behavioral pain scales, physiologic indicators are still lacking. In this presentation, recent evidence and limitations of vital signs’ inter-individual variability, pupil dilation reflex and cerebral measures (e.g., electroencephalograph, bispectral index) will first be discussed. The newly developed Nociception Level (NoL) index which simultaneously incorporates multiple physiologic parameters to estimate the pain level will then be introduced. Validation of this innovative technology has made progress in the field of anesthesia, and is recently being conducted in relation to procedural pain (i.e., chest tube removal/CTR) in the critical care setting.

In this presentation, preliminary results of the validation of the NoL index using a prospective cohort study design of cardiac surgery patients will be discussed. More specifically, these specific objectives will be addressed:

(1) Criterion validation of the NoL index with the patient’s self-report of pain intensity, pain unpleasantness and anxiety at rest and during CTR;

(2) Convergent validation of the NoL index with the patient’s behavioural responses during CTR;

(3) Discriminant validation of the NoL index between rest, a painful (i.e., CTR) and a non-painful (i.e., cuff inflation for blood pressure measurement) procedure.

Adequate pain assessment in patients who cannot communicate is critical if one wants to provide them with optimal analgesic treatment. Identification of valid physiological indicators of acute pain would represent a significant advance in our scientific knowledge for acute pain assessment in patients unable to self-report.

## Learning Objectives

1. To inform the audience about the psychometric properties of three scales measuring acute pain in children and commonly used in the pediatric emergency department (PED).

2. To inform the audience about the psychometric process associated with the testing and development of an electronic application associated with emotional and physical pain for Aboriginal children and youth.

3. To inform the audience about the validity of physiologic indicators (i.e., vital signs, pupil dilation reflex, cerebral measures) for the assessment of acute pain and to present preliminary data on the use of an innovative technology i.e., the Nociception Level Index.

## Coping with Pain: Basic, Developmental, and Translational Perspectives

Rebecca Pillai Riddell^a,*^, Loren Martin^b^, Lauren Campbell^c^, and Sylvie Le May^d^

^a^York University, Hospital for Sick Children and University of Toronto, Toronto, Canada; ^b^University of Toronto-Mississauga, Ontario, Canada; ^c^York University, Toronto, Ontario, Canada; ^d^Université de Montréal, Montreal, Ontario, Canada

**CONTACT** Rebecca Pillai Riddell rpr@yorku.ca


^*^Symposium Chair


© 2017 Rebecca Pillai Riddell, Loren Martin, Lauren Campbell, and Sylvie Le May. Published with license by Taylor & Francis.

This is an Open Access article distributed under the terms of the Creative Commons Attribution License (http://creativecommons.org/licenses/by/3.0/), which permits unrestricted use, distribution, and reproduction in any medium, provided the original work is properly cited. The moral rights of the named author(s) have been asserted.

Coping is considered a complex and dynamic process in which one’s thoughts and behaviors are continuously changing in response to specific demands appraised as stressful. Despite the importance of studying coping with pain, the question of how to define coping in this context has presented itself as a major issue in the field, with researchers exhibiting discrepant views on the operationalization of the coping construct. In the literature, the term ‘coping’ has been used to not only reflect behaviors that reduce distress but also to reflect the actual reduction of distress. It is both the outcome and the response by which that outcome is achieved.

After the chair provides a brief theoretical background to the study of coping and current challenges, this multidisciplinary presentation (with experts in neuroscience, developmental psychology and pediatric nursing) sets out to present new research on basic mechanisms of coping using animal models, the developmental of coping using longitudinal data of parent and children over the first five years of life, and a new clinical application for coping assessment with francophone adolescents after a major surgery. An audience-led discussion period, facilitated by the chair, will conclude the session.

## Speaker 1 Abstract Title: *Stress, pain and social interactions in mice*

**Speaker 1 Name**: Dr. Loren Martin

**Speaker 1 Affiliation**: Assistant Professor, University of Toronto- Mississauga, Ontario, Canada

**Speaker 1 Abstract**: Pain behaviors serve a communicative function in humans, and this may be true as well in other animals. Increasingly, data supports the ability of social contact, empathy, social buffering, and social stress to modulate pain sensitivity and pain behavior in mice and rats. In particular we find that familiarity between conspecifics is necessary for empathy behaviours and that increased stress hormones prevent the expression of these behaviours. Recently collected data indicates that the expression of empathy is prevented via an upregulation of the stress-related glucocorticoid receptor in distinct brain regions. Additionally, we have also developed novel paradigms for social reunion behaviours that modulate and are modulated by pain. We find that mice that have experienced pain display more social behaviours towards mice that are currently in pain. Pain experienced mice are more likely to groom, huddle and lick their cagemates when compared with a mouse that has not experienced the same pain stimulus. These models provide a new means for studying the relationship between social factors, stress and pain.

## Speaker 2 Abstract Title: *Parent and child predictors of children’s coping with needle-related procedures*

**Speaker 2 Name**: Ms. Lauren Campbell

**Speaker 2 Affiliation**: Doctoral Student, York University, Toronto, Ontario, Canada

**Speaker 2 Abstract**: A large-scale systematic review (Campbell, Pillai Riddell, et al., in press) recently examined the interrelationships between children’s coping responses, coping outcomes, and parent variables during needle-related procedures. Parent coping-promoting and distress-promoting behaviors enacted in combination were the most consistent predictors of optimal children’s coping responses, and less optimal children’s coping outcomes, respectively. Building on these findings, a recent study (Campbell, Pillai Riddell et al., submitted) considered child-related variables related to children’s coping with needle-related pain, in addition to parent variables. This study is the first of its kind to examine these relationships using a longitudinal design (i.e. following infants across the first year of life and then again at preschool). Four separate path models were estimated predicting children’s coping at the preschool immunization. Within the preschool appointment, findings indicated that combinations of parent coping- and distress-promoting behaviors did not predict subsequent children’s coping responses or coping outcomes. Most interestingly, these combinations of parent behaviors were related to concurrent children’s coping responses and outcomes. This suggests an important implication for parents to continuously engage in coping-promoting behaviors throughout the immunization appointment. Additionally, modeling suggested that parent sensitivity during the first year of life only predicted children’s coping verbalizations indirectly. Parental sensitivity predicted better developed children’s language abilities at preschool, which then related to increased rates of children’s coping verbalizations. These findings provide important and novel insights into the various developmental pathways predicting children’s coping with acute pain.

## Speaker 3 Abstract Title: *Validation of the French version of the Pain Coping Questionnaire (PCQ) with patients post-spinal fusion*

**Speaker 3 Name**: Dr. Sylvie Le May

**Speaker 3 Affiliation**: Associate Professor, Université de Montréal, Montreal, Ontario, Canada

**Speaker 3 Abstract**: Management of pain in adolescents with idiopathic scoliosis (AIS) who have undergone spinal fusion surgery is complex. Pain coping strategies combined with pharmacological approaches are essential to providing optimal pain management. Despite the importance of an individual’s coping strategies to rehabilitation, no validated translation of the Pain Coping Questionnaire (PCQ) is available for assessing French patients. The aim of this study was to translate the PCQ to French and validate the new measure (PCQ-F) using a sample of AIS post-spinal fusion patients. Content validity of the PCQ-F (39 items; Likert scale) was performed with patients and clinicians prior to pilot of the PCQ-F. We are currently recruiting patients who: 1) underwent a spinal fusion for their scoliosis, 2) are aged between 11 and 19 years, 3) read and write in French. Data collection is underway (111/195 patients recruited) and expected to be completed by March 2017. Preliminary psychometrics support the French translation of the PCQ in the post-surgical context. Final analyses will be presented based on internal consistency statistics (Cronbach’s Alphas), construct validity using Principal Component Analysis and correlations between socio-demographic variables and subfactors of the PCQ-F. A discussion of language nuances between French and English on the concept of coping will also be presented in the context of construct validity results. The goal of this study rests on the idea of better operationalizing coping strategies in different populations and cultures. It is envisioned that interventions could be developed according to idiosyncratic coping strategies identified as being helpful to manage a particular individual’s pain. Matching interventions to one’s coping strategies has the potential to decrease daily analgesics consumption and reduce side effects which would promote rehabilitation.

## Learning Objectives

1. Become versed in a novel animal paradigm that informs our understanding of how stress changes how the brain processes pain in ourselves and others.

2. Develop a grounding in developmental pathways of children’s coping with acute pain highlighting new data on the role of parent interactions and children’s language abilities.

3. Learn about a new version of a measure of pain coping that is based on state of the art data with French adolescents who have undergone spinal fusion surgery.

## Addressing the opioid crisis in Canada; who what where when why how

Fiona Campbell^a,*^, Paula Robeson^b^, and Jason W. Busse^c^

^a^Hospital for Sick Children, University of Toronto, President-elect Canadian Pain Society Toronto, Ontario, Canada; ^b^Strategic Partnerships and Knowledge Mobilization, Ottawa, Ontario, Canada; ^c^Department of Anesthesia, McMaster University, Hamilton, Ontario, Canada

**CONTACT** Fiona Campbell fiona.campbell@rogers.com


^*^Symposium Chair


© 2017 Fiona Campbell, Paula Robeson, and Jason W. Busse Published with license by Taylor & Francis

his is an Open Access article distributed under the terms of the Creative Commons Attribution License (http://creativecommons.org/licenses/by/3.0/), which permits unrestricted use, distribution, and reproduction in any medium, provided the original work is properly cited. The moral rights of the named author(s) have been asserted.

Canada faces a crisis with escalating, overdose and death caused by opioids. In response, the Federal and Ontario Ministers of Health, Jane Philpott and Eric Hoskins co-hosted a 2-day Opioid Conference and Summit in Ottawa, to develop a Joint Statement of Action to address this crisis. To quote Ministers Philpott and Hoskins “This is a complex health and social issue with devastating consequences for individuals, families, and communities. The response to this crisis needs to be comprehensive, collaborative, compassionate and evidence-based.”

The goals of this workshop are to provide an overview of i) the opioid crisis, ii) the Opioid Summit and the Joint Statement of Action committed by stakeholders, and iii) the updated Canadian Guideline for Safe and Effective Use of Opioids for Non-Cancer Pain, a key deliverable funded by Health Canada.

## Speaker 1 Abstrct Title: *Addressing the opioid crisis in Canada; Implications for the Canadian Pain Society and the pain community*

**Speaker 1 Name**: Fiona Campbell

**Speaker 1 Affiliation**: Hospital for Sick Children, University of Toronto, President-elect Canadian Pain Society Toronto, Ontario, Canada

**Speaker 1 Abstract**: The purpose of this presentation is to provide an overview of the opioid crisis leading to the Opioid Summit, and to describe what the Joint Statement of Action means for the Canadian Pain Society and people suffering with chronic pain. There are many contributory causes to this crisis including but not limited to illegally imported fentanyl, opioid overprescribing, lack of pharmacosurveillance, limited access to comprehensive addiction treatment programs, and poor pain management due in part to insufficient pain education, inadequate funding for pain research, and limited access to other treatment options. While opioid prescribing is cited as an important risk factor for the crisis, it is critical to distinguish between rates of illicit opioid use (increasing), and prescription opioid misuse (declining). In response to government pressure to curb opioid use there are disturbing trends emerging; physicians refusing to prescribe opioids fearing reprisal from professional bodies, suicides by pain patients for whom opioids were cut off, patients suffering from acute opioid withdrawal in ERs, and patients seeking illicit opioids to treat their pain. The CPS made specific commitments to the Joint Statement of Action including the need to promote i) improved access to multimodal pain treatments, ii) better pain education for health care providers and patients, and iii) that opioids remain available for those in whom they improve function and health related quality of life. Pain specific commitments to the Joint Statement of Action from other organizations will also be presented.

## Speaker 2 Abstract Title: *Addressing the opioid crisis in Canada: Progress on the Joint Statement of Action*

**Speaker 2 Name**: Paula Robeson

**Speaker 2 Affiliation**: Associate Director, Strategic Partnerships and Knowledge Mobilization, Ottawa, Ontario, Canada

**Speaker 2 Abstract**: The 2016 Opioid Summit co-hosted by the Honourable Jane Philpott, Federal Minister of Health and the Honourable Eric Hoskins, Ontario Minister of Health and Long-Term Care, provided an opportunity for experts and key decision-makers to establish a common understanding of the opioid crisis in Canada and commit to a joint approach to reduce opioid associated harms. Building upon the work already underway as part of the First Do No Harm strategy, the Canadian Centre on Substance Abuse provided backbone support to the organization, leadership, and coordination of the Summit and the Joint Statement of Action to Address the Opioid Crisis. CCSA is working with Health Canada in the facilitated follow-up with signatories to the Joint Statement of Action to engage and report on their commitments and their progress. The follow-up provides continued opportunities to share knowledge, seek feedback, discuss barriers, and exchange best practices to ensure the success of those commitments. The CCSA is also identifying and engaging new partners with clear accountability for action. This presentation will provide an overview of the Summit and discuss the progress and implementation of the Joint Statement of Action committed by stakeholders.

## Speaker 3 Abstract Title: *Addressing the opioid crisis in Canada; The new guideline for safe and effective use of opioids for non-cancer pain*

**Speaker 3 Name**: Jason W. Busse

**Speaker 3 Affiliation**: Department of Anesthesia, McMaster University, Hamilton, Ontario, Canada

**Speaker 3 Abstract**: This presentation will summarize the 2017 Canadian Guideline for opioids in the management of chronic non-cancer pain, funded by Health Canada. Each clinical practice recommendation as well the underlying evidence will be presented, together with the process for the development of recommendations. New considerations that informed the 2017 guideline (versus the 2010 guideline) will be discussed; specifically, use of the GRADE process to summarize quality of evidence, management of both financial and intellectual conflicts of interest, incorporation of patient values & preferences, and strategies to optimize the interpretation of evidence.

## Learning Objectives

1. Explain the opioid crisis that led to the Opioid Summit, and describe what the Joint Statement of Action means specifically for the Canadian Pain Society and people suffering with chronic pain.

2. Describe the progress and implementation of the Joint Statement of Action committed by stakeholders across Canada to address the opioid crisis.

3. Interpret statistically significant treatment effects of opioids for chronic non-cancer pain with respect to their clinical importance.

## Nice to meet you: How to present yourself and your ideas

Sarah Rosen^a,*^, Carley Ouellette^b,*^, Jeffrey Mogil^c^, Jack Hourigan, and Mario Di Carlo

^a^Integrated Program of Neuroscience, McGill University, Montreal, Canada; ^b^The Hospital for Sick Children, Toronto, Canada; ^c^Canada Research Chair in Genetics of Pain (Tier I) E. P. Taylor Chair in Pain Studies, Department of Psychology, McGill University, Montreal, Canada

**CONTACT** Sarah Rosen srosen625@gmail.com


^*^Symposium Chairs


© 2017 Sarah Rosen, Carley Ouellette, Jeffrey Mogil, Jack Hourigan, and Mario Di Carlo. Published with license by Taylor & Francis.

This is an Open Access article distributed under the terms of the Creative Commons Attribution License (http://creativecommons.org/licenses/by/3.0/), which permits unrestricted use, distribution, and reproduction in any medium, provided the original work is properly cited. The moral rights of the named author(s) have been asserted.

While dedication and hard work create a strong base for young scientists and professionals, refined communication skills are crucial for landing the “dream job”. Communication skills encompass a broad range of qualities such as: presenting information to large diverse audiences, sharing knowledge and expertise with colleagues and collaborators, and connecting with peers and patients. Strong communication and interpersonal skills are assets that contribute to success in academia, clinics, industry, and agencies, but require mindfulness and practice. This workshop will offer hands-on strategies on how to deliver an engaging presentation, how to build and stabilize professional relationships through networking, and how to improve your interpersonal skills in a range of settings.

## Speaker 1 Abstract Title: *How to give a better talk*

**Speaker 1 Name:** Jeffrey Mogil

**Speaker 1 Affiliation**: Canada Research Chair in Genetics of Pain (Tier I) E. P. Taylor Chair in Pain Studies, Department of Psychology, McGill University, Montreal, Canada

**Speaker 1 Abstract**: Verbal presentations of one’s work to various audiences represent a critical component of an academic career, yet scientists spend surprisingly little time thinking about and improving their talks. In a 20+ year career giving over 20 talks a year on average, I have concluded that most academic talks suffer from an erroneous starting assumption. This will be revealed, as will tips and tricks for making a more effective academic talk.

## Speaker 2 Abstract Title: *Having patience with patients*

**Speaker 2 Names:** Jack Hourigan and Mario Di Carlo

**Speaker 2 Abstract**: The ability to communicate effectively involves asking questions you may not have considered asking, and actively listening to the responses. In academia, although we often believe we are working to help others, we don’t always think about the others. This session will focus on the viewpoint of the pain patient. Jack Hourigan, the first parent to participate in the Family Integrated Care NICU study at Toronto’s Mount Sinai Hospital; and Mario Di Carlo, a chronic pain patient who is actively involved in chronic pain self-management programs, and patient engagement projects such as CIHR-SPOR, will talk about the goals and needs of a patient. This will be followed with an application phase, where the audience can put their knowledge into action.

## Speaker 3 Abstract Title: *Networking strategies*

**Speaker 3 Name:** Jack Hourigan

**Speaker 3 Abstract:** Strong communication skills often drive success. However, making connections and keeping connections is not as simple as it may sound. Anecdotal advice can be helpful, but the real way to develop communication skills is by setting goals and practicing. This session will provide an overview on successful networking techniques, followed by an opportunity to practice. This will be an interactive session with active participation from the audience.

## Learning Objectives

1. Learn how to organize and deliver a professional presentation with confidence.

2. Learn how to connect with, and communicate effectively, with colleagues, collaborators, and patients.

3. Develop networking skills through engagement and practice.

## A novel protocol of titrated, slow Ketamine infusions for CRPS and neuropathic pain: Rationale, outcomes, and behavioral correlates

Karen D. Davis 0000-0003-1879-0090 ^a,,b,*^, Ian Beauprie^c^, Anuj Bhatia^d^, and Rachael Bosma^e^

^a^Department of Surgery and Institute of Medical Science, University of Toronto, Toronto, Canada; ^b^Division of Brain, Imaging and Behaviour-Systems Neuroscience Krembil Research Institute Toronto Western Hospital, University Health Network; ^c^Department of Anesthesia, Pain Management, & Perioperative Medicine, Dalhousie University, Halifax, Nova Scotia, Canada; ^d^University of Toronto, Toronto, Ontario, Canada; ^e^Krembil Research Institute, University Health Network, Toronto, Ontario, Canada

**CONTACT** Karen D. Davis kdavis@uhnres.utoronto.ca


^*^Symposium Chair


© 2017 Karen D. Davis, Ian Beauprie, Anuj Bhatia, and Rachael Bosma. Published with license by Taylor & Francis.

This is an Open Access article distributed under the terms of the Creative Commons Attribution License (http://creativecommons.org/licenses/by/3.0/), which permits unrestricted use, distribution, and reproduction in any medium, provided the original work is properly cited. The moral rights of the named author(s) have been asserted.

Despite a plethora of treatment options available to treat complex regional pain syndrome (CRPS) and neuropathic pain (NP), up to two thirds of patients are refractory to treatment. Furthermore, treatments that do provide some effectiveness can be costly. Recent evidence suggests that low dose infusion of ketamine, an NMDA antagonist known for its anesthetic and depression-reliving properties, can provide significant relief of NP. However, its side effects, including psychedelic symptoms, can preclude its use. This symposium will 1) provide an overview of CRPS and NP, 2) provide insights into the bio-psycho-social aspects of living with relentless chronic pain and how life altering adequate treatment can be; as expressed from a first-person narrative provided by a patient with CRPS who has achieved pain relief from ketamine, 2) discuss the development of a low dose ketamine infusion protocol that is carefully titrated in each patient to minimize its negative side-effects while maximizing its long term analgesic properties, and 3) present behavioural data of individual differences in responders vs. non-responders to ketamine. Understanding baseline factors that can predict treatment response to ketamine will improve patient selection and protocol design for clinical studies.

## Speaker 1 Abstract Title: *Neuropathic Pain and CRPS: Scope of problem and existing treatments*

**Speaker 1 Name**: Ian Beauprie

**Speaker 1 Affiliation**: Associate Professor, Department of Anesthesia, Pain Management, & Perioperative Medicine, Dalhousie University, Halifax, Nova Scotia, Canada; Subspecialty Chief, Chronic Pain, Central Zone Nova Scotia Health Authority Chair, sub-specialty of Pain Medicine, Royal College of Physicians & Surgeons of Canada

**Speaker 1 Abstract**: Many cases of complex regional pain syndrome and neuropathic pain go on to be refractory and debilitating pain states. Existing evidence-based literature is limited, and such treatment often fails in clinical situations. Current treatments supported by evidence, guidelines or tradition will be reviewed.

## Speaker 2 Abstract Title: *IV ketamine infusions for CRPS and refractory neuropathic pain: Outcome data from over 5 years*

**Speaker 2 Name**: Anuj Bhatia

**Speaker 2 Affiliation**: Assistant Professor, University of Toronto, Toronto, Ontario, Canada; Director, Anesthesia Chronic Pain Clinical Services, Staff, Department of Anesthesia and Pain Management, University Health Network, Mount Sinai Hospital, and Women’s College Hospital, Toronto, Ontario, Canada; Chair, Special Interest Group on Neuropathic Pain of the Canadian Pain Society, Ontario representative, Pain Medicine Specialty Committee of the Royal College of Physicians and Surgeons of Canada, Ottawa, Ontario, Canada; Affiliated Faculty, Techna Institute for the Advancement of Technology for Health (Techna), Toronto, Ontario, Canada

**Speaker 2 Abstract**: Subanesthetic inpatient infusions of ketamine are a promising therapeutic option in the treatment of appropriately selected patients with refractory CRPS and neuropathic pain. The goal of our protocol is to effectively treat chronic pain while minimizing side effects. Our centre, Toronto Western Hospital, has been performing these infusions since 2010 with good outcomes in over 50% of patients. Data on the infusion protocol, possible factors influencing outcome, and adverse effects associated with this treatment will be presented.

## Speaker 3 Abstract Title: *Behavioural factors that contribute to the variability in treatment efficacy of low-dose ketamine infusion for neuropathic pain*

**Speaker 3 Name**: Rachael Bosma

**Speaker 3 Affiliation**: Postdoctoral Fellow; Krembil Research Institute, University Health Network, Toronto, ON, Canada

**Speaker 3 Abstract**: Individual factors may account, in part, for the variability in response to ketamine treatment for NP. Furthermore, the slow, low dose infusion of individually titrated ketamine infusion for NP is labour-intensive and costly. Thus, it is important to identify patients who would maximally benefit from this therapy. In this talk, I will present the results of quantitative sensory tests (including pain thresholds, temporal summation of pain, conditioned pain modulation) and questionnaires (including anxiety, depression, catastrophizing, and resilience) in patients with NP before and 1 month after treatment with ketamine. I will present 1) a characterization of the NP patients in contrast to healthy controls, 2) the non-specific effects of ketamine, and 3) the pain-specific effects of ketamine in NP patients.

## Learning Objectives

1. To understand the general characteristics of neuropathic pain and treatment options.

2. To be aware of a new protocol for IV ketamine infusions for refractory CRPS and neuropathic pain and of the potential life-changing outcomes, as shared from a patient perspective.

3. To be aware of the effects of ketamine on pain sensitivity, psychological and psychiatric measures, and of the pre-treatment characteristics of patients with NP who are responsive vs non-responsive to IV ketamine.

## New advances in cancer pain: Perspectives from across the lifespan

Paul Daeninck^a*^, Parker Murchison^b^, Christine Chambers^c^, and Perri Tutelman^c^

^a^St Boniface Unit and Attending Medical Oncologist, CancerCare Manitoba, Winnipeg, Manitoba, Canada; ^b^Halifax, Nova Scotia, Canada; ^c^Clinical Psychology at Dalhousie University and the IWK Health Centre, Halifax, Nova Scotia, Canada

**CONTACT** Paul Daeninckpdaeninck@cancercare.mb.ca


^*^Symposium Chair


© 2017 Paul Daeninck, Parker Murchison, Christine Chambers, and Perri Tutelman. Published with license by Taylor & Francis

This is an Open Access article distributed under the terms of the Creative Commons Attribution License (http://creativecommons.org/licenses/by/3.0/), which permits unrestricted use, distribution, and reproduction in any medium, provided the original work is properly cited. The moral rights of the named author(s) have been asserted.

Improvements in cancer survival have highlighted the importance of supportive care and symptom management across the disease trajectory. Cancer patients consistently rank pain as one of the most distressing symptoms associated with having cancer, citing impacts on quality of life, and physical and emotional functioning. The objective of the present workshop is to summarize advances in cancer pain across the lifespan in the context of research, practice, and firsthand patient experience. First, Parker Murchison, a 14- year-old cancer survivor, will describe his experiences with pain throughout his treatment for Acute Lymphoblastic Leukemia. Next, Perri Tutelman will present data from an online survey on the prevalence and characteristics of pediatric cancer pain around the world. Finally, Dr. Paul Daeninck will provide an overview of the new Canadian recommendations for the management of breakthrough cancer pain in adult patients. This research has relevance for clinicians and scientists working in various domains including psychosocial care and medical management. The information described in the current workshop will be presented in the context of how it may contribute to the development of new pain treatment protocols and psychosocial, family-centered interventions to ensure that all patients have access to optimal cancer-pain management.

## Speaker 1 Abstract Title: *Pain with acute lymphoblastic leukemia: A young patient’s experience*

**Speaker 1 Name**: Parker Murchison

**Speaker 1 Affiliation**: Halifax, Nova Scotia, Canada

**Speaker 1 Abstract**: At the age of 8, Parker was diagnosed with Acute Lymphoblastic Leukemia (ALL). Parker underwent treatment through the IWK Health Centre which consisted of daily oral chemotherapy and monthly spinal injections over the course of 3.5 years. Now 14, Parker is cancer free, and recalls the pain he experienced as a difficult part of his treatment. In his presentation, Parker will describe his personal experiences with pain as an ALL patient and survivor. He will discuss the sources and characteristics of his pain during treatment, the impact it had on him and his family, and what strategies he found helpful in managing it. Parker will conclude with what, in his experience, clinicians and researchers can do to help support children and their families with cancer pain.

## Speaker 2 Abstract Title: *Prevalence, characteristics, and management of pain in children with cancer*

**Speaker 2 Name**: Perri Tutelman

**Speaker 2 Affiliation**: PhD student in Clinical Psychology at Dalhousie University and the IWK Health Centre, Halifax, Nova Scotia, Canada

**Speaker 2 Abstract**: Each year, approximately 200,000 children and adolescents are diagnosed with cancer worldwide. Shifts towards outpatient models of cancer care have made parents increasingly responsible for assessing and treating their child’s cancer pain at home from diagnosis through survivorship. However, literature on what parents do to manage their children’s cancer pain is scarce. Perri will present new data from a study examining the prevalence and characteristics of pediatric cancer pain and parents’ experiences with cancer pain management. 230 parents (89% mothers) of children (50% male, mean age=8.93 years, sd=4.50 years) with various cancer types currently in treatment, remission, or who are survivors completed an online survey. Parents from 12 countries participated (50% from Canada), with over 65% reporting that their child experienced pain within the past month. The most common source of pain was treatment-related (46%), followed by everyday pains (25%), and procedural pain (21%). Parents reported using both medications and non-pharmacological techniques to manage their child’s pain. Parents’ confidence in managing their child’s cancer pain varied as a function of their perception of their child’s pain. This study highlights the significant burden of pain experienced by children with cancer, and the pain management strategies parents are using at home to manage it. The implications of these findings for future research and the development of family-centered interventions for pediatric cancer pain management will be discussed.

## Speaker 3 Abstract Title: *Breakthrough cancer pain: Canadian recommendations for proper management*

**Speaker 3 Name**: Paul Daeninck

**Speaker 3 Affiliation**: Site Coordinator, St Boniface Unit and Attending Medical Oncologist, CancerCare Manitoba, Winnipeg, Manitoba, Canada; Assistant Professor, Dept of Internal Medicine, Cross appointment to Dept of Family Medicine, Max Rady College of Medicine, University of Manitoba, Winnipeg, Manitoba, Canada; Palliative Medicine Consultant, Winnipeg Regional Health Authority Palliative Care Program

**Speaker 3 Abstract**: Pain is one of the most common and, indeed, most feared consequences of cancer. Proper assessment and appropriate treatment strategies can help alleviate the associated physical, emotional and psychosocial impacts for people with cancer. Breakthrough cancer pain (BTcP) represents a relatively recently identified element of the spectrum of pain affecting cancer patients. In Canada, guidelines for cancer pain management have not included uniform recommendations for the management of BTcP, contributing to the regional variability in its management. In this current climate of concern about the rational use of opioids for pain control, recommendations can help guide health care professionals in their choice of therapy. In 2015, a group of Canadian specialists in palliative care, oncology and anaesthesiology developed a set of evidence-based recommendations to assist Canadian health care providers in the assessment and treatment of BTcP. In this workshop, the now published recommendations will be reviewed, and the use of novel transmucosal fentanyl formulations as well as non-pharmacologic options will be discussed as part of a comprehensive approach to BTcP.

## Learning Objectives

1. To consider the experience of cancer pain from a patient’s perspective.

2. To describe the prevalence and characteristics of pediatric cancer pain and parents’ cancer-pain management practices

3. To review the Canadian recommendations for the management of breakthrough cancer pain and integrate them in a comprehensive plan to treat pain in adults with cancer

## Who is teaching whom about pain management? Novel training and mentoring initiatives engaging patients and trainees

Bonnie B.,^*^^1^^2^, Judy J.^1^, Michael M.^3^, David K. D. K.^4^, Carley C.^5^

^a^Lawrence S Bloomberg Faculty of Nursing, University of Toronto, Toronto, Ontario, Canada; ^b^The Hospital for Sick Children, Toronto, Ontario, Canada; ^c^McMaster University, Hamilton, Ontario, Canada; ^d^Faculty of Dentistry, University of Toronto, Toronto, Ontario, Canada; ^e^Western University, London, Ontario, Canada

**CONTACT** Bonnie Stevens b.stevens@utoronto.ca


^*^Symposium Chair


© 2017 Bonnie Stevens, Judy Watt-Watson, Michael McGillion, David K. Lam, and Carley Ouellette. Published with license by Taylor & Francis.

This is an Open Access article distributed under the terms of the Creative Commons Attribution License (http://creativecommons.org/licenses/by/3.0/), which permits unrestricted use, distribution, and reproduction in any medium, provided the original work is properly cited. The moral rights of the named author(s) have been asserted.

Patients continue to rate their pain management as suboptimal in clinical practice. To address this issue, there is an urgent need to ensure that patients’ voices are heard in health care professional training initiatives on pain. Developing pain management competencies requires a comprehensive training approach that has been outlined in the IASP curriculum. Key components of this curriculum include 4 consensus-derived competencies categorized within the domains of the multidimensional nature of pain, pain assessment and measurement, management of pain, and context of pain management. These domains capture the complexity of pain assessment and management across the life span in the context of various settings, populations, and care team models. This approach optimally encompasses strategic training at the undergraduate and pre-licensure level but can also be applied across the continuum of professional development. To enhance pain management practices, and clinical and organizational outcomes, we need to create a critical mass of health care professionals competent in pain assessment and management. Each of the presenters will focus on the development, implementation and evaluation of their unique training initiatives and use this as a platform for discussion for evaluating pain training programs with a particular eye on their success in engaging patients and trainees.

## Speaker 1 Abstract Title: *Putting patients at the centre: A Pre-Licensure interprofessional pain education program*

**Speaker 1 Name**: Bonnie Stevens

**Speaker 1 Affiliation**: Lawrence S Bloomberg Faculty of Nursing, University of Toronto; The Hospital for Sick Children, Toronto, Ontario, Canada

**Speaker 1 Abstract**: The University of Toronto Centre for the Study of Pain (UTCSP) has developed and implemented the internationally-recognized and award-winning Inter-Faculty Pain Curriculum (IPC). The IPC is a 20-hour, inter-professional pain curriculum that engages patients in (a) large group sessions (e.g. patient panel), concurrent patient-centered research-based sessions, facilitated small groups who use patient-oriented case studies, and self-study modules. All learning activities involve trainees from multiple disciplines working together to critically appraise current basic science and clinical evidence and apply it to the development of inter-professional patient-based pain assessment and management plans. Since 2002, approximately 1000 trainees from 7 different disciplines have completed the IPC each year, for a cumulative total of approximately 15,000 trainees. The IPC consistently demonstrates statistically significant improvements in pain knowledge and beliefs (~12% increase in 2015 across faculties) as well as high levels of satisfaction with the interprofessional learning experience (satisfaction with the patient panel rates most highly in terms of satisfaction; ~90% are extremely satisfied). New strategies to promote patient engagement in learning include online learning modules (e.g. on opioid use and its impact on patients), integration of real-time social media directed at patients and a novel inter-professional formative evaluation strategy that focuses on individual application of pain knowledge to patients. Results of over a decade of IPC evaluation will be presented, with the aim of determining new approaches to enhancing and engaging patient-engagement and improvement in pain competencies.

## Speaker 2 Abstract Title: *A patient-focused innovative web-based education model: The pain education interprofessional resource (PEIR)*

**Speaker 2 Name**: Judy Watt-Watson (1) and Michael McGillion (2)

**Speaker 2 Affiliation**: (1) Lawrence S. Bloomberg Faculty of Nursing, University of Toronto, Toronto, Ontario, Canada; (2) McMaster University, Hamilton, Ontario, Canada

**Speaker 2 Abstract**: Although the prevalence of unrelieved pain cannot be solved by a single profession, there are very few models of interprofessional pain education, particularly at the pre-licensure level. Also, it is essential that our students value patient stories and can collaborate with colleagues as they integrate what they are being taught in real world contexts. While web-based innovations are pervasive in education, there has been minimal evaluation of such models in the context of pain education. Funded by CIHR, we developed and piloted an innovative, web-based, Pain Education Interprofessional Resource (PEIR) (developers: Watt-Watson, McGillion, Lax, Oskarsson, Hunter), based upon the IASP pain curriculum and related core competencies. PEIR consists of three modules that follow a surgical patient and her family trying to manage acute to persistent postoperative pain one year later. Additional simulated patient scenarios are included that illustrate key common clinician misbeliefs in practice. Interactive learning technologies are used throughout that include video simulations, contextualized resources, and narrated commentaries to promote critical appraisal and application of research to complex patient pain problems. Both formative and summative evaluation methods included the Past Assessment Skills Tool (PAST) to examine pain assessment, interpersonal skill and empathy. Students’ overall usability scores were very positive 4.27/5 (SD 0.56) and there was a 20% improvement in pain knowledge following PEIR (p<0.001).

## Speaker 3 Abstract Title: *Engaging patients with pain in summertime learning: The connaught summer institute in pain*

**Speaker 3 Name**: (1) David K. Lam and (2) Carley Ouellette

**Speaker 3 Affiliation**: (1) Faculty of Dentistry, University of Toronto, Toronto, Ontario, Canada; (2) Western University, London, Ontario, Canada

**Speaker 3 Abstract**: Pain is an elusive universal phenomenon that health professionals across all disciplines encounter in individuals at all ages, in all health care settings and across all situations. We are at a critical juncture where research of pain mechanisms and pain management strategies require effective translation to ensure optimal outcomes for individuals who suffer from pain. The goal of the Connaught Summer Institute in Pain, an annual 4-day high-intensity workshop that culminates with a 1-day international scientific meeting, is to bring together graduate students, postdoctoral fellows, clinicians and experts in both basic science and clinical fields from leading international universities, to foster interdisciplinary collaboration and creative new methods for pain education and research. Engaging patients in the Institute is crucial since a key aspect of the Institute is to stimulate and support the development of research questions based on clinical issues that health professionals encounter in daily practice. The inaugural 2016 Institute engaged various patients with 37 graduate students, postdoctoral fellows, and clinicians. There were 18 Presentations with 18 speakers, including patients, clinicians, clinical scientists, and basic scientists; 2 interactive knowledge translation sessions; and 3 Panel Discussions with Patients/Clinicians/Scientists. Participant feedback was extremely positive: 100% agreed/strongly agreed that the Patient Panels stimulated their thinking in these areas and were overall well done; 95% agreed/strongly agreed that they were satisfied with their learning experience about pain; and 90% agreed/strongly agreed that they intend to apply the knowledge and skills they acquired in their work and would recommend the Institute to their peers.

## Learning Objectives

1. Explore opportunities for engaging patients in innovative training opportunities to improve pain knowledge and competencies as outlined in the IASP curriculum.

2. Determine strategies to foster strong linkages between trainees, patients and mentors to enhance interdisciplinary education and research collaboration, innovation and trainee success.

3. Describe the value of diverse and unique partnership experiences and engaging partnerships across research settings, clinics, training networks, centers and stakeholders.

